# Multi-attribute decision-making method based on q-rung orthopair probabilistic hesitant fuzzy schweizer-sklar power weighted hamy mean operator

**DOI:** 10.1371/journal.pone.0266779

**Published:** 2023-02-15

**Authors:** Zhiyuan Chen, Di Shen, Fuping Yu, Xinlei Tang, Zhe Zhang

**Affiliations:** Air Traffic Control and Navigation College, Air Force Engineering University, Xi’an, Shaanxi Province, China; Hangzhou Normal University, CHINA

## Abstract

In order to further improve the computing power of the information aggregation operator in the q-rung orthopair probabilistic hesitant fuzzy environment, this paper proposes a multi-attribute decision-making method based on the q-rung orthopair probabilistic hesitant fuzzy Schweizer-Sklar power weighted Hamy mean operator. Firstly, the algorithm of q-rung orthopair probabilistic hesitant fuzzy set is improved based on the Schweizer-Sklar T-norm. In order to better reflect the degree of hesitation of decision-making experts, a new q-rung orthopair probabilistic hesitant fuzzy distance measure is proposed, which provides a basis for subsequent power weighted calculations. Furthermore, considering the correlation between attributes and the influence of data extremes, some information aggregation operators and their power weighted forms are proposed. Finally, a multi-attribute decision-making model based on the q-rung orthopair probabilistic hesitant fuzzy Schweizer-Sklar power weighted Hamy mean operator is established, and the reliability and validity of the research content in this paper are verified through decision-making examples and comparative analysis.

## 1. Introduction

Multi-attribute decision-making refers to the process in which a decision-making expert evaluates a limited set of alternatives from multiple aspects, and comprehensively weighs the evaluation information to determine the best solution. At present, the multi-attribute decision-making theory is widely used in medical diagnosis [1], supplier selection [2], power distribution network optimization [3] and other issues. However, in practical applications, it is difficult to quantify some of the attribute indicators used to evaluate the scheme, decision-making expert can only give qualitative evaluations, and cannot give an accurate value to express their own evaluation information. In addition, subject to the different levels of cognitive abilities of decision-making expert, their evaluation of alternatives will inevitably have certain uncertainty.

In order to describe the evaluation information of decision-making expert more accurately, Zadeh proposed fuzzy set [4]. Fuzzy set use membership functions to indicate the degree of membership of elements to the set, which provides a good method and idea for dealing with multi-attribute decision-making problems. On this basis, many experts and scholars have enriched and expanded the fuzzy set theory, many types of fuzzy set theories have been proposed successively [5–8]. Since this kind of fuzzy set can only describe the membership degree of a single element, it has certain limitations. Therefore, Atanassov proposed the intuitionistic fuzzy set [9], which describes the evaluation information more comprehensively by introducing the degree of membership, the degree of non-membership, and the degree of hesitation. But the intuitionistic fuzzy set needs to satisfy the constraint that the sum of the degree of membership and the degree of non-membership is less than or equal to 1. For this reason, Yager proposed the Pythagorean fuzzy set [10], and broadened its constraint condition to the extent that the sum of the square of the degree of membership and the degree of non-membership is less than or equal to 1, which can describe the evaluation information more widely. Based on the advantages of Pythagorean hesitation fuzzy sets, many experts and scholars have carried out exploration and promotion, and successively proposed q-rung orthopair fuzzy set [11], interval-valued q-rung orthopair fuzzy set [12], Pythagorean hesitant fuzzy set [13] and q-rung orthopair hesitant fuzzy set [14]. These theories further broaden the description scope of evaluation information and provide new tools and methods for solving multi-attribute decision-making problems. In practical applications, different decision-making experts have different levels of knowledge, and the importance of evaluation information given by them is also different. In order to solve such problems, Xu’s team took the lead in combining probability information with the above theories and proposed probability hesitant fuzzy set [15,16]. Subsequently, Liu et al. [17] proposed probabilistic hesitant fuzzy cumulative residual entropy and combined it with maximum entropy to obtain the incomplete probabilities of the probabilistic hesitant fuzzy element, and developed an emergency group decision-making method. Garg et al. [18] further generalized the probabilistic hesitant fuzzy set from the perspective of membership and non-membership, and proposed probabilistic dual hesitant fuzzy set. Ren et al. [19] broadens the value range of membership degree and non-membership degree, and proposed q-rung orthopair probabilistic hesitant fuzzy set (q-ROPHFS). Expressing the preference of decision-making expert through probability information can more truly reflect the importance of the decision-making opinions given by the decision-making expert, and at the same time, it can describe more decision-making information of the decision makers in actual situations. Therefore, it has become a hot spot in the current research field.

In order to obtain a clear and intuitive multi-attribute decision-making result, the evaluation information of multiple decision-making experts needs to be fused into a comprehensive evaluation value, which can provide a reference for the subsequent selection of the optimal scheme. With its unique advantages, information aggregation operator can fuse multi-dimensional fuzzy information into a single overall value, and become a commonly used information fusion tool in the process of multi-attribute decision-making. Currently commonly used information aggregation operators are ordered weighted average operator [20], generalized ordered weighted average operator [21] and induced ordered weighted average operator [22]. These operators all start from the weighted average of the data and consider the ranking of the evaluation information in the process of information aggregation. In order to avoid the decision result from being affected by the subjective evaluation of the decision-making expert, Yager proposed the power average operator [23]. It constructs the weight by constructing the support relationship between a single data and all other data, so that the process of information aggregation becomes objective. In practical applications, there is often a certain correlation between attribute indicators, which requires the information aggregation operator to be able to capture this information during the process of information aggregation. Based on this part, experts and scholars use choquet integral aggregation operator [24], bonferroni mean operator [25], heronian mean operator [26], maclaurin symmetric mean operator [27], and muirhead mean operator [28] for the aggregation of various fuzzy information, at the same time, the problem of information fusion in multi-attribute decision-making is explored. In order to further improve the information aggregation ability under the correlation of attribute indicators, Hara proposed the Hamy mean operator [29], which more flexibly considers the relationship between multiple attribute indicators and has stronger functions. In addition, in order to further improve the capabilities of information aggregation operators, some experts and scholars have improved the operators based on Schweizer-Sklar T-norm [30], Frank norm [31] and other T-norms, making the improved information integration operators more flexible.

In summary, although the research on multi-attribute decision-making based on q-ROPHFS has become a hot spot and has achieved certain results, the research on its information aggregation operator is still in its infancy. In order to better aggregate the evaluation information of decision-making expert and improve the computing power of information aggregation operator, this paper proposes a multi-attribute decision-making method based on the q-rung orthopair probabilistic hesitant fuzzy Schweizer-Sklar power weighted Hamy average operator. Firstly, the algorithm of q-rung orthopair probabilistic hesitant fuzzy set based on Schweizer-Sklar T-norm is defined, and a new distance measure is proposed to provide the basis for subsequent multi-attribute decision-making operations. Then, the q-rung orthopair probabilistic hesitant fuzzy Schweizer-Sklar average (q-ROPHFSSA) operator and the q-rung orthopair probabilistic hesitant fuzzy Schweizer-Sklar geometric (q-ROPHFSSG) operator are respectively defined. On this basis, considering the impact of the correlation between attributes in practical applications on the decision results, the q-rung orthopair probabilistic hesitant fuzzy Schweizer-Sklar Hamy mean (q-ROPHFSSHM) operator and the q-rung orthopair probabilistic hesitant fuzzy Schweizer-Sklar double Hamy mean (q-ROPHFSSDHM) operator, the relevant calculation formula is given, its related properties are studied, and the special form of the operator is discussed. At the same time, considering the influence of the importance of attributes and the support relationship, the q-rung orthopair probabilistic hesitant fuzzy Schweizer-Sklar power weighted Hamy mean (q-ROPHFSSPWHM) operator and the q-rung orthopair probabilistic hesitant fuzzy Schweizer-Sklar power weighted double Hamy mean (q-ROPHFSSPWDHM) operator are defined. Finally, a multi-attribute decision-making model based on the q-ROPHFSSPWHM operator is established to solve the problem of air traffic control sectors evaluation. The reliability and validity of the research content in this paper are verified through decision-making examples and comparative analysis.

## 2. Basic knowledge

### 2.1 The basic definition of q-rung orthopair probabilistic hesitant fuzzy set

**Definition 1.** [19] Let *X* ={*x*_1_,*x*_2_,⋯,*x*_*n*_} be a given universe, then the q-order orthogonal probability hesitant fuzzy set on the universe of q-ROPHFS is defined as:

$$A=\{<x,\Gamma_{A}(x),\Psi_{A}(x){>}_{q}|x\in X\}$$

Where $\Gamma_{A}(x)=\{\mu_{1}(p_{1}),\mu_{2}(p_{2}),\cdots ,\mu_{l}(p_{l})\}$, $\Psi_{A}(x)=\{\nu_{1}({\bar{p}}_{1}),\nu_{2}({\bar{p}}_{2}),\cdots ,\nu_{m}({\bar{p}}_{m})\}$, *μ*_*l*_ and *ν*_*m*_ respectively represent the possible membership and non-membership of element *x*∈*X*, and *p*_*l*_ and ${\bar{p}}_{m}$ represent the corresponding probability. For ∀*x*∈*X*, ∀*μ*∈Γ_*A*_(*X*) and ∀*ν*∈Ψ_*A*_(*X*), the following conditions are all satisfied:

$$0<\mu ,\nu <1,\;0\leq \mu^{q}+\nu^{q}\leq 1,\;0<p_{l},{\bar{p}}_{m}<1,\;\sum_{l=1}^{\left|\Gamma_{A}(x)\right|}p_{l}\leq 1,\sum_{m=1}^{\left|\Psi_{A}(x)\right|}{\bar{p}}_{m}\leq 1$$

Where |Γ_*A*_(*x*)| and |Ψ_*A*_(*x*)| represent the number of elements contained in Γ_*A*_(*x*) and Ψ_*A*_(*x*) respectively. Call <Γ_*A*_(*x*),Ψ_*A*_(*x*)>_*q*_ q-rung orthopair probabilistic hesitatnt fuzzy element (q-ROPHFE), denoted as *h* = <Γ_*A*_(*x*),Ψ_*A*_(*x*)>_*q*_.

**Definition 2.** [19] Let *h* = <Γ_*h*_,Ψ_*h*_>_*q*_, $h_{1}=<\Gamma_{h_{1}},\Psi_{h_{1}}{>}_{q}$, $h_{2}=<\Gamma_{h_{2}},\Psi_{h_{2}}{>}_{q}$ be any three q-ROPHFE, and *λ*>0, then the algorithm of q-ROPHFS ics as follows:

$$h_{1}⊕h_{2}=\langle \underset{\begin{array}{l} \mu_{1_{l}}\in \Gamma_{h_{1}}\\ \mu_{2_{k}}\in \Gamma_{h_{2}} \end{array}}{\cup }\{\left[{\left(\mu_{1_{l}}^{q}+\mu_{2_{k}}^{q}-\mu_{1_{l}}^{q}\mu_{2_{k}}^{q}\right]}^{\frac{1}{q}})(\frac{p_{1_{l}}p_{2_{k}}}{\sum_{l=1}^{\left|\Gamma_{h_{1}}\right|}p_{1_{l}}\sum_{k=1}^{\left|\Gamma_{h_{2}}\right|}p_{2_{k}}}\right)\},{\underset{\begin{array}{l} \nu_{1_{m}}\in \Psi_{h_{1}}\\ \nu_{2_{n}}\in \Psi_{h_{2}} \end{array}}{\cup }\{\left[\nu_{1_{m}}\nu_{2_{n}}](\frac{{\bar{p}}_{1_{m}}{\bar{p}}_{2_{n}}}{\sum_{m=1}^{\left|\Psi_{h_{1}}\right|}{\bar{p}}_{1_{m}}\sum_{n=1}^{\left|\Psi_{h_{2}}\right|}{\bar{p}}_{2_{n}}}\right)\}\rangle }_{q}$$ (1)

$$h_{1}⊗h_{2}=\langle \underset{\begin{array}{l} \mu_{1_{l}}\in \Gamma_{h_{1}}\\ \mu_{2_{k}}\in \Gamma_{h_{2}} \end{array}}{\cup }\{\left[\mu_{1_{l}}\mu_{2_{k}}](\frac{p_{1_{l}}p_{2_{k}}}{\sum_{l=1}^{\left|\Gamma_{h_{1}}\right|}p_{1_{l}}\sum_{k=1}^{\left|\Gamma_{h_{2}}\right|}p_{2_{k}}}\right)\},{\underset{\begin{array}{l} \nu_{1_{m}}\in \Psi_{h_{1}}\\ \nu_{2_{n}}\in \Psi_{h_{2}} \end{array}}{\cup }\{\left[{\left(\nu_{1_{m}}^{q}+\nu_{2_{n}}^{q}-\nu_{1_{m}}^{q}\nu_{2_{n}}^{q}\right)}^{{}^{\frac{1}{q}}}](\frac{{\bar{p}}_{1_{m}}{\bar{p}}_{2_{n}}}{\sum_{m=1}^{\left|\Psi_{h_{1}}\right|}{\bar{p}}_{1_{m}}\sum_{n=1}^{\left|\Psi_{h_{2}}\right|}{\bar{p}}_{2_{n}}}\right)\}\rangle }_{q}$$ (2)

$$\lambda h={\langle \underset{\mu_{l}\in \Gamma_{h}}{\cup }\{\left[{\left(1-{\left(1-\mu_{l}^{q}\right)}^{\lambda }\right)}^{\frac{1}{q}}](p_{l}\right)\},\underset{\nu_{m}\in \Psi_{h}}{\cup }\{\nu_{m}^{\lambda }({\bar{p}}_{m})\}\rangle }_{q}$$ (3)

$$h^{\lambda }={\langle \underset{\mu_{l}\in \Gamma_{h}}{\cup }\{\mu_{l}^{\lambda }(p_{l})\},\underset{\nu_{m}\in \Psi_{h}}{\cup }\{\left[{\left(1-{\left(1-\nu_{m}^{q}\right)}^{\lambda }\right)}^{\frac{1}{q}}]({\bar{p}}_{m}\right)\}\rangle }_{q}$$ (4)

$$h^{c}={\langle \Psi_{h},\Gamma_{h}\rangle }_{q}$$ (5)

**Definition 3.** [19] Let *h* = <Γ_*h*_,Ψ_*h*_>_*q*_ be a q-ROPHFE, then the score function *S*(*h*) is defined as:

$$S(h)=\frac{1}{2}(\frac{\sum_{l=1}^{\left|\Gamma_{h}\right|}\mu_{l}^{q}\cdot p_{l}}{\sum_{l=1}^{\left|\Gamma_{h}\right|}p_{l}}+\frac{\sum_{m=1}^{\left|\Psi_{h}\right|}(1-v_{m}^{q})\cdot {\bar{p}}_{m}}{\sum_{m=1}^{\left|\Psi_{h}\right|}{\bar{p}}_{m}})$$

Where |Γ_*h*_| and |Ψ_*h*_| represent the number of elements contained in Γ_*h*_ and Ψ_*h*_ respectively. Let *h*_1_ and *h*_2_ be two q-ROPHFE, if *S*(*h*_1_)>*S*(*h*_2_), then *h*_1_ is better than *h*_2_, denoted as *h*_1_≻*h*_2_. If *S*(*h*_1_) = *S*(*h*_2_), it cannot be compared by the score function *S*(*h*).

**Definition 4.** [19] Let *h* = <Γ_*h*_,Ψ_*h*_>_*q*_ be a q-ROPHFE, its scoring function is *η*, then the deviation degree *D*(*h*) is defined as:

$$D(h)=\frac{1}{2}(\frac{\sum_{l=1}^{\left|\Gamma_{h}\right|}p_{l}{\left(\mu_{l}^{q}-S{\left(h\right)}^{q}\right)}^{2}}{\sum_{l=1}^{\left|\Gamma_{h}\right|}p_{l}}+\frac{\sum_{m=1}^{\left|\Psi_{h}\right|}{\bar{p}}_{m}{\left((1-v_{m}^{q})-S{\left(h\right)}^{q}\right)}^{2}}{\sum_{m=1}^{\left|\Psi_{h}\right|}{\bar{p}}_{m}})$$

The comparison rules corresponding to the q-ROPHFE based on the score function and the deviation degree are as follows:

If *S*(*h*_1_)>*S*(*h*_2_), then *h*_1_≻*h*_2_.

If *S*(*h*_1_) = *S*(*h*_2_),

2.1) If *D*(*h*_1_)>*D*(*h*_2_), then *h*_1_≺*h*_2_.

2.2) If *D*(*h*_1_) = *D*(*h*_2_), then *h*_1_ = *h*_2_.

### 2.2 Schweizer-Sklar norm

The T-norm is a binary function with a value on [0,1], which has important application value in the fields of probability metric space, decision theory, statistics, games, functional equations, etc.

**Definition 5.** [32] Let T be a binary operation on [0,1], *T*:[0,1]^2^→[0,1], any *x*,*y*,*z*∈[0,1], if the following conditions are met:

T(x,y)=T(y,x) (6)

T(x,T(y,z))=T(T(x,y),z) (7)

wheny≤z,T(x,y)≤T(x,z) (8)

T(x,1)=x (9)

Then T is said to be a T-norm. Let S be a binary operation on [0,1], *S*:[0,1]^2^→[0,1], any *x*,*y*,*z*∈[0,1], if it satisfies (1), (2), (3), and satisfies *S*(*x*,0) = *x*, then S is a T-norm.

The Schweizer-Sklar norm is a special T-norm, which has good flexibility and can make the information integration operator have good robustness.

**Definition 6.** [33] Schweizer-Sklar T-norm is usually expressed as:

$$T_{SS,r}={\left(x^{r}+y^{r}-1\right)}^{\frac{1}{r}}$$

$$T_{SS,r}^{*}=1-{\left({\left(1-x\right)}^{r}+{\left(1-y\right)}^{r}-1\right)}^{\frac{1}{r}}$$

Where *r*<0, *x*,*y*∈[0,1], *T*_*SS*,*r*_ represent Schweizer-Sklar T-norm, and $T_{SS,r}^{*}$ represents Schweizer-Sklar T-conorm. When *r* = 0, the Schweizer-Sklar norm degenerates to a simple algebraic norm as follows:

$$T_{r}\left(x,y\right)=xy$$

$$T_{r}^{*}(x,y)=x+y-xy$$

### 2.3 Algorithm of q-rung orthopair probabilistic hesitant fuzzy set based on Schweizer-Sklar T-norm

In order to solve the problem of insufficient flexibility of the Muirhead mean operator mentioned in document [19], a new q-ROPHFS algorithm is defined based on the Schweizer-Sklar T-norm, which provides a basis for subsequent multi-attribute decision-making applications. Here, based on the operation of Schweizer-Sklar T-norm, the algorithm of q-ROPHFS is redefined.

**Definition 7.** Let *h* = <Γ_*h*_,Ψ_*h*_>_*q*_, $h_{1}=<\Gamma_{h_{1}},\Psi_{h_{1}}{>}_{q}$, $h_{2}=<\Gamma_{h_{2}},\Psi_{h_{2}}{>}_{q}$ be any three q-ROPHFE and *λ*>0, then the algorithm for the q-ROPHFS based on Schweizer-Sklar T-norm is as follows:

$$h_{1}⊕h_{2}={\langle \underset{\begin{array}{l} \mu_{1_{l}}\in \Gamma_{h_{1}}\\ \mu_{2_{k}}\in \Gamma_{h_{2}} \end{array}}{\cup }\{\left[{\left(1-{\left({\left(1-\mu_{1_{l}}^{q}\right)}^{r}+{\left(1-\mu_{2_{k}}^{q}\right)}^{r}-1\right)}^{\frac{1}{r}}\right)}^{\frac{1}{q}}](\frac{p_{1_{l}}p_{2_{k}}}{\sum_{l=1}^{\left|\Gamma_{h_{1}}\right|}p_{1_{l}}\sum_{k=1}^{\left|\Gamma_{h_{2}}\right|}p_{2_{k}}}\right)\},\underset{\begin{array}{l} \nu_{1_{m}}\in \Psi_{h_{1}}\\ \nu_{2_{n}}\in \Psi_{h_{2}} \end{array}}{\cup }\{\left[{\left({\left(\nu_{1_{m}}^{q}\right)}^{r}+{\left(\nu_{2_{n}}^{q}\right)}^{r}-1\right)}^{\frac{1}{qr}}](\frac{{\bar{p}}_{1_{m}}{\bar{p}}_{2_{n}}}{\sum_{m=1}^{\left|\Psi_{h_{1}}\right|}{\bar{p}}_{1_{m}}\sum_{n=1}^{\left|\Psi_{h_{2}}\right|}{\bar{p}}_{2_{n}}}\right)\}\rangle }_{q}$$ (10)

$$h_{1}⊗h_{2}={\langle \underset{\begin{array}{l} \mu_{1_{l}}\in \Gamma_{h_{1}}\\ \mu_{2_{k}}\in \Gamma_{h_{2}} \end{array}}{\cup }\{\left[{\left({\left(\mu_{1_{l}}^{q}\right)}^{r}+{\left(\mu_{2_{k}}^{q}\right)}^{r}-1\right)}^{\frac{1}{qr}}](\frac{p_{1_{l}}p_{2_{k}}}{\sum_{l=1}^{\left|\Gamma_{h_{1}}\right|}p_{1_{l}}\sum_{k=1}^{\left|\Gamma_{h_{2}}\right|}p_{2_{k}}}\right)\},\underset{\begin{array}{l} \nu_{1_{m}}\in \Psi_{h_{1}}\\ \nu_{2_{n}}\in \Psi_{h_{2}} \end{array}}{\cup }\{\left[{\left(1-{\left({\left(1-\nu_{1_{m}}^{q}\right)}^{r}+{\left(1-\nu_{2_{n}}^{q}\right)}^{r}-1\right)}^{\frac{1}{r}}\right)}^{\frac{1}{q}}](\frac{{\bar{p}}_{1_{m}}{\bar{p}}_{2_{n}}}{\sum_{m=1}^{\left|\Psi_{h_{1}}\right|}{\bar{p}}_{1_{m}}\sum_{n=1}^{\left|\Psi_{h_{2}}\right|}{\bar{p}}_{2_{n}}}\right)\}\rangle }_{q}$$ (11)

$$\lambda h={\langle \underset{\mu_{l}\in \Gamma_{h}}{\cup }\{\left[{\left(1-{\left(\lambda {\left(1-\mu_{l}^{q}\right)}^{r}-(\lambda -1)\right)}^{\frac{1}{r}}\right)}^{\frac{1}{q}}](p_{l}\right)\},\underset{\nu_{m}\in \Psi_{h}}{\cup }\{\left[{\left(\lambda \nu_{m}^{qr}-(\lambda -1)\right)}^{\frac{1}{qr}}]({\bar{p}}_{m}\right)\}\rangle }_{q}$$ (12)

$$h^{\lambda }={\langle \underset{\mu_{l}\in \Gamma_{h}}{\cup }\{\left[{\left(\lambda \mu_{l}^{qr}-(\lambda -1)\right)}^{\frac{1}{qr}}](p_{l}\right)\},\underset{\nu_{m}\in \Psi_{h}}{\cup }\{\left[{\left(1-{\left(\lambda {\left(1-\nu_{m}^{q}\right)}^{r}-(\lambda -1)\right)}^{\frac{1}{r}}\right)}^{\frac{1}{q}}]({\bar{p}}_{m}\right)\}\rangle }_{q}$$ (13)

$$h^{c}={\langle \Psi_{h},\Gamma_{h}\rangle }_{q}$$ (14)

The algorithm of q-ROPHFS improved based on Schweizer-Sklar T-norm also satisfies certain operational properties, see Theorem 1:

**Theorem 1.** Let *h* = <Γ_*h*_,Ψ_*h*_>_*q*_, $h_{1}=<\Gamma_{h_{1}},\Psi_{h_{1}}{>}_{q}$, $h_{2}=<\Gamma_{h_{2}},\Psi_{h_{2}}{>}_{q}$ be any three q-ROPHFE and *λ*>0, then:

$$h_{1}⊕h_{2}=h_{2}⊕h_{1}$$ (15)

$$h_{1}⊗h_{2}=h_{2}⊗h_{1}$$ (16)

$$\lambda (h_{1}⊕h_{2})=\lambda h_{1}⊕\lambda h_{2}$$ (17)

$${\left(h_{1}⊗h_{2}\right)}^{\lambda }=h_{1}{}^{\lambda }⊗h_{2}{}^{\lambda }$$ (18)

$$(\lambda_{1}+\lambda_{2})h=\lambda_{1}h⊕\lambda_{2}h$$ (19)

$$h^{\left(\lambda_{1}+\lambda_{2}\right)}=h^{\lambda_{1}}⊗h^{\lambda_{2}}$$ (20)

**Prove:** It is easy to prove that (1) and (2) are established by definition 7. The following proves that (3) holds:

Step 1:

$$\begin{array}{l} \lambda (h_{1}⊕h_{2})=\langle \underset{\begin{array}{l} \mu_{1_{l}}\in \Gamma_{h_{1}}\\ \mu_{2_{k}}\in \Gamma_{h_{2}} \end{array}}{\cup }\{\left[{\left(1-{\left(\lambda {\left(1-\mu_{1_{l}}^{q}\right)}^{r}+\lambda {\left(1-\mu_{2_{k}}^{q}\right)}^{r}-(2\lambda -1)\right)}^{\frac{1}{r}}\right)}^{\frac{1}{q}}](\frac{p_{1_{l}}p_{2_{k}}}{\sum_{l=1}^{\left|\Gamma_{h_{1}}\right|}p_{1_{l}}\sum_{k=1}^{\left|\Gamma_{h_{2}}\right|}p_{2_{k}}}\right)\},\\ \;{\underset{\begin{array}{l} \nu_{1_{m}}\in \Psi_{h_{1}}\\ \nu_{2_{n}}\in \Psi_{h_{2}} \end{array}}{\cup }\{\left[{\left(\lambda {\left(\nu_{1_{m}}^{q}\right)}^{r}+\lambda {\left(\nu_{2_{n}}^{q}\right)}^{r}-(2\lambda -1)\right)}^{\frac{1}{qr}}](\frac{{\bar{p}}_{1_{m}}{\bar{p}}_{2_{n}}}{\sum_{m=1}^{\left|\Psi_{h_{1}}\right|}{\bar{p}}_{1_{m}}\sum_{n=1}^{\left|\Psi_{h_{2}}\right|}{\bar{p}}_{2_{n}}}\right)\}\rangle }_{q} \end{array}$$

Step 2:

$$\begin{array}{l} \lambda h_{1}=\\ {\langle \underset{\mu_{1_{l}}\in \Gamma_{h_{1}}}{\cup }\{\left[{\left(1-{\left(\lambda {\left(1-\mu_{1_{l}}^{q}\right)}^{r}-(\lambda -1)\right)}^{\frac{1}{r}}\right)}^{\frac{1}{q}}](p_{l}\right)\},\underset{\nu_{1_{m}}\in \Psi_{h_{1}}}{\cup }\{\left[{\left(\lambda {\left(\nu_{1_{m}}^{q}\right)}^{r}-(\lambda -1)\right)}^{\frac{1}{qr}}]({\bar{p}}_{m}\right)\}\rangle }_{q} \end{array}$$

Step 3:

$$\begin{array}{l} \lambda h_{2}=\\ {\langle \underset{\mu_{2_{k}}\in \Gamma_{h_{2}}}{\cup }\{\left[{\left(1-{\left(\lambda {\left(1-\mu_{2_{k}}^{q}\right)}^{r}-(\lambda -1)\right)}^{\frac{1}{r}}\right)}^{\frac{1}{q}}](p_{k}\right)\},\underset{\nu_{2_{n}}\in \Psi_{h_{2}}}{\cup }\{\left[{\left(\lambda {\left(\nu_{2_{n}}^{q}\right)}^{r}-(\lambda -1)\right)}^{\frac{1}{qr}}]({\bar{p}}_{n}\right)\}\rangle }_{q} \end{array}$$

Step 4:

$$\begin{array}{l} \lambda h_{1}⊕\lambda h_{2}=\langle \underset{\begin{array}{l} \mu_{1_{l}}\in \Gamma_{h_{1}}\\ \mu_{2_{k}}\in \Gamma_{h_{2}} \end{array}}{\cup }\{\left[{\left(1-{\left(\lambda {\left(1-\mu_{1_{l}}^{q}\right)}^{r}+\lambda {\left(1-\mu_{2_{k}}^{q}\right)}^{r}-(2\lambda -1)\right)}^{\frac{1}{r}}\right)}^{\frac{1}{q}}](\frac{p_{1_{l}}p_{2_{k}}}{\sum_{l=1}^{\left|\Gamma_{h_{1}}\right|}p_{1_{l}}\sum_{k=1}^{\left|\Gamma_{h_{2}}\right|}p_{2_{k}}}\right)\},\\ {\;\underset{\begin{array}{l} \nu_{1_{m}}\in \Psi_{h_{1}}\\ \nu_{2_{n}}\in \Psi_{h_{2}} \end{array}}{\cup }\{\left[{\left(\lambda {\left(\nu_{1_{m}}^{q}\right)}^{r}+\lambda {\left(\nu_{2_{n}}^{q}\right)}^{r}-(2\lambda -1)\right)}^{\frac{1}{qr}}](\frac{{\bar{p}}_{1_{m}}{\bar{p}}_{2_{n}}}{\sum_{m=1}^{\left|\Psi_{h_{1}}\right|}{\bar{p}}_{1_{m}}\sum_{n=1}^{\left|\Psi_{h_{2}}\right|}{\bar{p}}_{2_{n}}}\right)\}\rangle }_{q} \end{array}$$

Therefore, (3) is proved to be true, and (4) is proved by the same reason.

The following proves that (5) holds:

Step 1:

$$\begin{array}{l} (\lambda_{1}+\lambda_{2})h=\langle \underset{\mu_{l}\in \Gamma_{h}}{\cup }\{\left[{\left(1-{\left(\left(\lambda_{1}+\lambda_{2}){\left(1-\mu_{l}^{q}\right)}^{r}-(\lambda_{1}+\lambda_{2}-1\right)\right)}^{\frac{1}{r}}\right)}^{\frac{1}{q}}](p_{l}\right)\},\\ {\;\underset{\nu_{m}\in \Psi_{h}}{\cup }\{\left[{\left(\left(\lambda_{1}+\lambda_{2})\nu_{m}^{qr}-(\lambda_{1}+\lambda_{2}-1\right)\right)}^{\frac{1}{qr}}]({\bar{p}}_{m}\right)\}\rangle }_{q} \end{array}$$

Step 2:

$$\lambda_{1}h={\langle \underset{\mu_{l}\in \Gamma_{h}}{\cup }\{\left[{\left(1-{\left(\lambda_{1}{\left(1-\mu_{l}^{q}\right)}^{r}-(\lambda_{1}-1)\right)}^{\frac{1}{r}}\right)}^{\frac{1}{q}}](p_{l}\right)\},\underset{\nu_{m}\in \Psi_{h}}{\cup }\{\left[{\left(\lambda_{1}\nu_{m}^{qr}-(\lambda_{1}-1)\right)}^{\frac{1}{qr}}]({\bar{p}}_{m}\right)\}\rangle }_{q}$$

Step 3:

$$\lambda_{2}h={\langle \underset{\mu_{l}\in \Gamma_{h}}{\cup }\{\left[{\left(1-{\left(\lambda_{2}{\left(1-\mu_{l}^{q}\right)}^{r}-(\lambda_{2}-1)\right)}^{\frac{1}{r}}\right)}^{\frac{1}{q}}](p_{l}\right)\},\underset{\nu_{m}\in \Psi_{h}}{\cup }\{\left[{\left(\lambda_{2}\nu_{m}^{qr}-(\lambda_{2}-1)\right)}^{\frac{1}{qr}}]({\bar{p}}_{m}\right)\}\rangle }_{q}$$

Step 4:

$$\begin{array}{l} \lambda_{1}h⊕\lambda_{2}h=\langle \underset{\mu_{l}\in \Gamma_{h}}{\cup }\{\left[{\left(1-{\left(\left(\lambda_{1}+\lambda_{2}){\left(1-\mu_{l}^{q}\right)}^{r}-(\lambda_{1}+\lambda_{2}-1\right)\right)}^{\frac{1}{r}}\right)}^{\frac{1}{q}}](p_{l}\right)\},\\ \;{\underset{\nu_{m}\in \Psi_{h}}{\cup }\{\left[{\left(\left(\lambda_{1}+\lambda_{2})\nu_{m}^{qr}-(\lambda_{1}+\lambda_{2}-1\right)\right)}^{\frac{1}{qr}}]({\bar{p}}_{m}\right)\}\rangle }_{q} \end{array}$$

Therefore, (5) is proved to be true, and (6) is proved by the same reason.

### 2.4 Hamy mean operator

**Definition 8.** [34] Let *h*_*i*_(*i* = 1,2,⋯,*n*) denote the set of values, and *k* = 1,2,⋯,*n*, if:

$$HM^{\left(k\right)}(h_{1},h_{2},\cdots ,h_{n})=\frac{\sum_{1\leq i_{1}\leq \cdots \leq i_{n}}{\left(\prod_{j=1}^{k}a_{i_{j}}\right)}^{\frac{1}{k}}}{C_{n}^{k}}$$

Then call *HM*^(*k*)^ the Hamy mean, where (*i*_1_,*i*_2_,⋯,*i*_*k*_) represents all k-element combinations traversing (1,2,⋯,*n*), and $C_{n}^{k}$ represents the binomial coefficient.

## 3 Improved q-rung orthopair probabilistic hesitant fuzzy distance measure

q-ROPHFE contains a number of different degrees of membership and non-membership, reflecting the differences between decision-making experts, and reflect the degree of hesitation among decision-making experts. When the probabilities corresponding to different degrees of membership and non-membership are closer, the more difficult it is for decision-making experts to reach consensus and the higher the degree of hesitation. Based on this, this paper defines the hesitation degree of the q-ROPHFE.

**Definition 9.** Let *h* = <Γ_*h*_,Ψ_*h*_>_*q*_ be a q-ROPHFE, the hesitation *H*(*h*) is defined as:

$$H(h)=\frac{1}{2}(\frac{\sum_{l=1}^{\left|\Gamma_{h}\right|}|p_{l}-\frac{1}{\left|\Gamma_{h}\right|}\sum_{i=1}^{\left|\Gamma_{h}\right|}p_{i}|{\left(\mu_{l}^{q}-S{\left(h\right)}^{q}\right)}^{2}}{\sum_{l=1}^{\left|\Gamma_{h}\right|}p_{l}}+\frac{\sum_{m=1}^{\left|\Psi_{h}\right|}|{\bar{p}}_{m}-\frac{1}{\left|\Psi_{h}\right|}\sum_{j=1}^{\left|\Psi_{h}\right|}{\bar{p}}_{j}|{\left((1-v_{m}^{q})-S{\left(h\right)}^{q}\right)}^{2}}{\sum_{m=1}^{\left|\Psi_{h}\right|}{\bar{p}}_{m}})$$

In order to fully reflect the deviation between decision-making experts, this article defines a distance of q-ROPHFE based on the degree of hesitation.

**Theorem 2.** Let *h*_1_ and *h*_2_ be two q-ROPHFE, then the distance of q-ROPHFE based on hesitation is:

$$d(h_{1},h_{2})=\tilde{\alpha }{\left(\frac{1}{2}\sum_{i=1}^{l_{\Gamma }}p^{*}{\left|\left({\left(\mu_{1}^{\theta (i)}\right)}^{q}-{\left(\mu_{2}^{\theta (i)}\right)}^{q}\right)\right|}^{\lambda }+\frac{1}{2}\sum_{j=1}^{l_{\Psi }}{\bar{p}}^{*}{\left|\left({\left(\nu_{1}^{\theta (j)}\right)}^{q}-{\left(\nu_{2}^{\theta (j)}\right)}^{q}\right)\right|}^{\lambda }\right)}^{\frac{1}{\lambda }}+\tilde{\beta }|H(h_{1})-H(h_{2})|$$

Where *λ* is the power exponent, which is selected according to the actual situation, $\tilde{\alpha }$ and $\tilde{\beta }$ represent the balance coefficient, satisfy $\tilde{\alpha }+\tilde{\beta }=1$, $\tilde{\alpha },\tilde{\beta }\in [0,1]$, usually take 0.5. $p^{*}$ and ${\bar{p}}^{*}$ are the maximum values when $p_{1}^{\sigma (i)}/p^{*}$, $p_{2}^{\sigma (i)}/p^{*}$ and ${\bar{p}}_{1}^{\sigma (i)}/{\bar{p}}^{*}$, ${\bar{p}}_{2}^{\sigma (i)}/{\bar{p}}^{*}$ are positive integers respectively. For $\forall \mu_{1}^{\sigma (i)}(p_{1}^{\sigma (i)})\in \Gamma_{h_{1}}$, if there is $p_{1}^{\sigma (1)}/p^{*}=m$, then there is $\mu_{1}^{\sigma (1)}=\mu_{1}^{\theta (1)}=\mu_{1}^{\theta (2)}=\cdots =\mu_{1}^{\theta (m)}$. If there is $p_{1}^{\sigma (2)}/p^{*}=n$, then there is $\mu_{1}^{\sigma (2)}=\mu_{1}^{\theta (m+1)}=\mu_{1}^{\theta (m+2)}=\cdots =\mu_{1}^{\theta (m+n)}$. $\mu_{1}^{\sigma (i)}$ is the i-th largest value in $\Gamma_{h_{1}}$ and satisfies $\mu_{1}^{\sigma (1)}>\mu_{1}^{\sigma (2)}>\cdots >\mu_{1}^{\sigma (i)}>\cdots >\mu_{1}^{\sigma (|\Gamma_{h_{1}}|)}$. Similarly, when *i* = 1,2,⋯,*l*_Γ_, *j* = 1,2,⋯,*l*_Ψ_, the above-mentioned corresponding relationship exists for $\forall \mu_{1}^{\theta (i)}$, $\forall \mu_{2}^{\theta (i)}$, $\forall \nu_{1}^{\theta (j)}$ and $\forall \nu_{2}^{\theta (j)}$. In practical problems, the sum of the probability information of *h*_1_ and *h*_2_ is usually not equal, so it is necessary to add elements to make the sum of the probability information of both equal. This article assumes that decision-making expert are risk-averse, so the element with the smallest value is added.

## 4 q-rung orthopair probabilistic hesitant fuzzy Schweizer-Sklar T-norm aggregation operator

This section defines the q-ROPHFSSA operator, the q-ROPHFSSG operator, the q-ROPHFSSHM operator and the q-ROPHFSSDHM operator. The related calculation formulas are given, the related properties are studied, and the special forms of operators are discussed, which provides a basis for the subsequent use of Hamy mean operator for multi-attribute decision-making modeling.

### 4.1 q-rung orthopair probabilistic hesitant fuzzy Schweizer-Sklar average operator

**Definition 10.** Let $h_{i}=<\Gamma_{h_{i}},\Psi_{h_{i}}{>}_{q}(i=1,2,\cdots .n)$ be a set of q-ROPHFE, then the q-ROPHFSSA operator is defined as:

$$q-ROPHFSSA(h_{1},h_{2},\cdots ,h_{n})=\frac{1}{n}\overset{n}{\underset{i=1}{⊕}}h_{i}$$

**Theorem 3.** Let $h_{i}=<\Gamma_{h_{i}},\Psi_{h_{i}}{>}_{q}(i=1,2,\cdots .n)$ be a set of q-ROPHFE, then the result of using q-ROPHFSSA operator to aggregate information is still the q-ROPHFE, and satisfies:

$$\begin{array}{l} q-ROPHFSSA(h_{1},h_{2},\cdots ,h_{n})=\\ {\langle \underset{\begin{array}{c} \mu_{i_{l}}\in \Gamma_{h_{i}}\\ i=1,2,\cdots n \end{array}}{\cup }\{\left[{\left(1-{\left(\frac{1}{n}\sum_{i=1}^{n}{\left(1-\mu_{i_{l}}^{q}\right)}^{r}\right)}^{\frac{1}{r}}\right)}^{\frac{1}{q}}](\prod_{i=1}^{n}\frac{p_{i_{l}}}{\sum_{l=1}^{\left|\Gamma_{h_{i}}\right|}p_{i_{l}}}\right)\},\underset{\begin{array}{l} \nu_{i_{m}}\in \Psi_{h_{i}}\\ i=1,2,\cdots n \end{array}}{\cup }\{\left[{\left(\frac{1}{n}\sum_{i=1}^{n}\nu_{i_{m}}^{qr}\right)}^{\frac{1}{qr}}](\prod_{i=1}^{n}\frac{{\bar{p}}_{i_{m}}}{\sum_{m=1}^{\left|\Psi_{h_{i}}\right|}{\bar{p}}_{i_{m}}}\right)\}\rangle }_{q} \end{array}$$

**Prove:** Firstly, use mathematical induction to prove:

$$\overset{n}{\underset{i=1}{⊕}}h_{i}={\langle \underset{\begin{array}{c} \mu_{i_{l}}\in \Gamma_{h_{i}}\\ i=1,2,\cdots n \end{array}}{\cup }\{\left[{\left(1-{\left(\sum_{i=1}^{n}\left({\left(1-\mu_{i_{l}}^{q}\right)}^{r}-1\right)+1\right)}^{\frac{1}{r}}\right)}^{\frac{1}{q}}](\prod_{i=1}^{n}\frac{p_{i_{l}}}{\sum_{l=1}^{\left|\Gamma_{h_{i}}\right|}p_{i_{l}}}\right)\},\underset{\begin{array}{l} \nu_{i_{m}}\in \Psi_{h_{i}}\\ i=1,2,\cdots n \end{array}}{\cup }\{\left[{\left(\sum_{i=1}^{n}\left(\nu_{i_{m}}^{qr}-1\right)+1\right)}^{\frac{1}{qr}}](\prod_{i=1}^{n}\frac{{\bar{p}}_{i_{m}}}{\sum_{m=1}^{\left|\Psi_{h_{i}}\right|}{\bar{p}}_{i_{m}}}\right)\}\rangle }_{q}$$

When *n* = 2,

$$\begin{array}{l} \overset{2}{\underset{i=1}{⊕}}h_{i}=h_{1}⊕h_{2}\\ ={\langle \underset{\begin{array}{l} \mu_{1_{l}}\in \Gamma_{h_{1}}\\ \mu_{2_{l}}\in \Gamma_{h_{2}} \end{array}}{\cup }\{\left[{\left(1-{\left({\left(1-\mu_{1_{l}}^{q}\right)}^{r}+{\left(1-\mu_{2_{l}}^{q}\right)}^{r}-1\right)}^{\frac{1}{r}}\right)}^{\frac{1}{q}}](\frac{p_{1_{l}}p_{2_{l}}}{\sum_{l=1}^{\left|\Gamma_{h_{1}}\right|}p_{1_{l}}\sum_{l=1}^{\left|\Gamma_{h_{2}}\right|}p_{2_{l}}}\right)\},\underset{\begin{array}{l} \nu_{1_{m}}\in \Psi_{h_{1}}\\ \nu_{2_{m}}\in \Psi_{h_{2}} \end{array}}{\cup }\{\left[{\left({\left(\nu_{1_{m}}^{q}\right)}^{r}+{\left(\nu_{2_{m}}^{q}\right)}^{r}-1\right)}^{\frac{1}{qr}}](\frac{{\bar{p}}_{1_{m}}{\bar{p}}_{2_{m}}}{\sum_{m=1}^{\left|\Psi_{h_{1}}\right|}{\bar{p}}_{1_{m}}\sum_{m=1}^{\left|\Psi_{h_{2}}\right|}{\bar{p}}_{2_{m}}}\right)\}\rangle }_{q}\\ ={\langle \underset{\begin{array}{c} \mu_{i_{l}}\in \Gamma_{h_{i}}\\ i=1,2,\cdots n \end{array}}{\cup }\{\left[{\left(1-{\left(\sum_{i=1}^{2}\left({\left(1-\mu_{i_{l}}^{q}\right)}^{r}-1\right)+1\right)}^{\frac{1}{r}}\right)}^{\frac{1}{q}}](\prod_{i=1}^{2}\frac{p_{i_{l}}}{\sum_{l=1}^{\left|\Gamma_{h_{i}}\right|}p_{i_{l}}}\right)\},\underset{\begin{array}{l} \nu_{i_{m}}\in \Psi_{h_{i}}\\ i=1,2,\cdots n \end{array}}{\cup }\{\left[{\left(\sum_{i=1}^{2}\left(\nu_{i_{m}}^{qr}-1\right)+1\right)}^{\frac{1}{qr}}](\prod_{i=1}^{2}\frac{{\bar{p}}_{i_{m}}}{\sum_{m=1}^{\left|\Psi_{h_{i}}\right|}{\bar{p}}_{i_{m}}}\right)\}\rangle }_{q} \end{array}$$

Therefore, the equation holds when *n* = 2. Assuming that *n* = *k* holds, that is:

$$\overset{k}{\underset{i=1}{⊕}}h_{i}={\langle \underset{\begin{array}{c} \mu_{i_{l}}\in \Gamma_{h_{i}}\\ i=1,2,\cdots k \end{array}}{\cup }\{\left[{\left(1-{\left(\sum_{i=1}^{k}\left({\left(1-\mu_{i_{l}}^{q}\right)}^{r}-1\right)+1\right)}^{\frac{1}{r}}\right)}^{\frac{1}{q}}](\prod_{i=1}^{k}\frac{p_{i_{l}}}{\sum_{l=1}^{\left|\Gamma_{h_{i}}\right|}p_{i_{l}}}\right)\},\underset{\begin{array}{l} \nu_{i_{m}}\in \Psi_{h_{i}}\\ i=1,2,\cdots k \end{array}}{\cup }\{\left[{\left(\sum_{i=1}^{k}\left(\nu_{i_{m}}^{qr}-1\right)+1\right)}^{\frac{1}{qr}}](\prod_{i=1}^{k}\frac{{\bar{p}}_{i_{m}}}{\sum_{m=1}^{\left|\Psi_{h_{i}}\right|}{\bar{p}}_{i_{m}}}\right)\}\rangle }_{q}$$

When *n* = *k*+1,

$$\begin{array}{l} \overset{k+1}{\underset{i=1}{⊕}}h_{i}=(\overset{k}{\underset{i=1}{⊕}}h_{i})⊕h_{k+1}=\langle \underset{\begin{array}{c} \mu_{i_{l}}\in \Gamma_{h_{i}}\\ i=1,2,\cdots k+1 \end{array}}{\cup }\{\left[{\left(1-{\left(\sum_{i=1}^{k}\left({\left(1-\mu_{i_{l}}^{q}\right)}^{r}-1\right)+1+{\left(1-\mu_{k+1_{l}}^{q}\right)}^{r}-1\right)}^{\frac{1}{r}}\right)}^{\frac{1}{q}}](\prod_{i=1}^{k}\frac{p_{i_{l}}}{\sum_{l=1}^{\left|\Gamma_{h_{i}}\right|}p_{i_{l}}}\cdot \frac{p_{k+1_{l}}}{\sum_{l=1}^{\left|\Gamma_{h_{i}}\right|}p_{{}_{l+1}}}\right)\},\\ {\;\underset{\begin{array}{l} \nu_{i_{m}}\in \Psi_{h_{i}}\\ i=1,2,\cdots k+1 \end{array}}{\cup }\{\left[{\left(\sum_{i=1}^{k}\left(\nu_{i_{m}}^{qr}-1\right)+1+\nu_{k+1_{m}}^{qr}-1\right)}^{\frac{1}{qr}}](\prod_{i=1}^{k}\frac{{\bar{p}}_{i_{m}}}{\sum_{m=1}^{\left|\Psi_{h_{i}}\right|}{\bar{p}}_{i_{m}}}\cdot \frac{{\bar{p}}_{k+1_{m}}}{\sum_{m=1}^{\left|\Psi_{h_{i}}\right|}{\bar{p}}_{k+1_{m}}}\right)\}\rangle }_{q}\\ \;={\langle \underset{\begin{array}{c} \mu_{i_{l}}\in \Gamma_{h_{i}}\\ i=1,2,\cdots k+1 \end{array}}{\cup }\{\left[{\left(1-{\left(\sum_{i=1}^{k+1}\left({\left(1-\mu_{i_{l}}^{q}\right)}^{r}-1\right)+1\right)}^{\frac{1}{r}}\right)}^{\frac{1}{q}}](\prod_{i=1}^{k+1}\frac{p_{i_{l}}}{\sum_{l=1}^{\left|\Gamma_{h_{i}}\right|}p_{i_{l}}}\right)\},\underset{\begin{array}{l} \nu_{i_{m}}\in \Psi_{h_{i}}\\ i=1,2,\cdots k+1 \end{array}}{\cup }\{\left[{\left(\sum_{i=1}^{k+1}\left(\nu_{i_{m}}^{qr}-1\right)+1\right)}^{\frac{1}{qr}}](\prod_{i=1}^{k+1}\frac{{\bar{p}}_{i_{m}}}{\sum_{m=1}^{\left|\Psi_{h_{i}}\right|}{\bar{p}}_{i_{m}}}\right)\}\rangle }_{q} \end{array}$$

Therefore, the equation holds when *n* = *k*+1, that is, the equation holds for *i* = 1,2,⋯*n*. From definition 7, we can get:

$$\frac{1}{n}\overset{n}{\underset{i=1}{⊕}}h_{i}={\langle \underset{\begin{array}{c} \mu_{i_{l}}\in \Gamma_{h_{i}}\\ i=1,2,\cdots n \end{array}}{\cup }\{\left[{\left(1-{\left(\frac{1}{n}\sum_{i=1}^{n}{\left(1-\mu_{i_{l}}^{q}\right)}^{r}\right)}^{\frac{1}{r}}\right]}^{\frac{1}{q}})(\prod_{i=1}^{n}\frac{p_{i_{l}}}{\sum_{l=1}^{\left|\Gamma_{h_{i}}\right|}p_{i_{l}}}\right)\},\underset{\begin{array}{l} \nu_{i_{m}}\in \Psi_{h_{i}}\\ i=1,2,\cdots n \end{array}}{\cup }\{\left[{\left(\frac{1}{n}\sum_{i=1}^{n}\nu_{i_{m}}^{qr}\right)}^{\frac{1}{qr}}](\prod_{i=1}^{n}\frac{{\bar{p}}_{i_{m}}}{\sum_{m=1}^{\left|\Psi_{h_{i}}\right|}{\bar{p}}_{i_{m}}}\right)\}\rangle }_{q}$$

That is, theorem 2 is proved.

The q-ROPHFSSA operator has the following properties:

**Theorem 4. (Idempotence)** Let $h_{i}=<\Gamma_{h_{i}},\Psi_{h_{i}}{>}_{q}(i=1,2,\cdots .n)$ be a set of q-ROPHFE, if there is *h*_*i*_ = *h* for any *i*(*i* = 1,2,⋯.*n*), then:

$$q-ROPHFSSA(h_{1},h_{2},\cdots ,h_{n})=h$$

**Theorem 5. (Monotonicity)** Let $h_{i}=<\Gamma_{h_{i}},\Psi_{h_{i}}{>}_{q}(i=1,2,\cdots .n)$, $h_{i}^{\prime }=<\Gamma_{h_{i}^{\prime }},\Psi_{h_{i}^{\prime }}{>}_{q}(i=1,2,\cdots .n)$ be two sets of q-ROPHFE, if there is $h_{i}<h_{i}^{\prime }$ for any *i*(*i* = 1,2,⋯.*n*), then:

$$q-ROPHFSSA(h_{1},h_{2},\cdots ,h_{n})\leq q-ROPHFSSA(h_{1}^{\prime },h_{2}^{\prime },\cdots ,h_{n}^{\prime })$$

**Theorem 6. (Boundedness)** Let $h_{i}=<\Gamma_{h_{i}},\Psi_{h_{i}}{>}_{q}(i=1,2,\cdots .n)$ be a set of q-ROPHFE, $h^{-}=<\Gamma_{h}^{-},\Psi_{h}^{+}{>}_{q}$, $h^{+}=<\Gamma_{h}^{+},\Psi_{h}^{-}{>}_{q}$, where *μ*^−^ = min(*μ*∈Γ_*i*_}, *μ*^+^ = max(*μ*∈Γ_*i*_}, *ν*^−^ = min(*ν*∈Ψ_*i*_}, *ν*^+^ = max(*ν*∈Ψ_*i*_}, then:

$$h^{-}\leq q-ROPHFSSA(h_{1},h_{2},\cdots ,h_{n})\leq h^{+}$$

**Theorem 7. (Permutation invariance)** Let $h_{i}=<\Gamma_{h_{i}},\Psi_{h_{i}}{>}_{q}(i=1,2,\cdots .n)$ be a set of q-ROPHFE, For any permutation of *h*_*i*_(*i*=1,2,⋯.*n*) and $h_{i}^{\prime }(i=1,2,\cdots .n)$, there are:

$$q-ROPHFSSA(h_{1},h_{2},\cdots ,h_{n})=q-ROPHFSSA(h_{1}^{\prime },h_{2}^{\prime },\cdots ,h_{n}^{\prime })$$

**Definition 11.** Let $h_{i}=<\Gamma_{h_{i}},\Psi_{h_{i}}{>}_{q}(i=1,2,\cdots .n)$ be a set of q-ROPHFE, the weight vector is ***ω*** = (*ω*_1_,*ω*_2_,⋯,*ω*_*n*_)^*T*^, and satisfies $\sum_{i=1}^{n}\omega_{i}=1$ and *ω*_*i*_∈[0,1], then the q-rung orthopair probabilistic hesitant fuzzy Schweizer-Sklar weighted average (q-ROPHFSSWA) operator is defined as:

$$q-ROPHFSSWA(h_{1},h_{2},\cdots ,h_{n})=\frac{1}{n}\overset{n}{\underset{i=1}{⊕}}\omega_{i}h_{i}$$

**Theorem 8.** Let $h_{i}=<\Gamma_{h_{i}},\Psi_{h_{i}}{>}_{q}(i=1,2,\cdots .n)$ be a set of q-ROPHFE, the weight vector is ***ω*** = (*ω*_1_,*ω*_2_,⋯,*ω*_*n*_)^*T*^, and satisfies $\sum_{i=1}^{n}\omega_{i}=1$ and *ω*_*i*_∈[0,1], then the result of using q-ROPHFSSWA operator to aggregate information is still the q-ROPHFE, and satisfies:

$$\begin{array}{l} q-ROPHFSSWA(h_{1},h_{2},\cdots ,h_{n})=\\ {\langle \underset{\begin{array}{c} \mu_{i_{l}}\in \Gamma_{h_{i}}\\ i=1,2,\cdots n \end{array}}{\cup }\{\left[{\left(1-{\left(\frac{1}{n}\sum_{i=1}^{n}\left(\omega_{i}{\left(1-\mu_{i_{l}}^{q}\right)}^{r}-\omega_{i}+1\right)\right)}^{\frac{1}{r}}\right)}^{\frac{1}{q}}](\prod_{i=1}^{n}\frac{p_{i_{l}}}{\sum_{l=1}^{\left|\Gamma_{h_{i}}\right|}p_{i_{l}}}\right)\},\underset{\begin{array}{l} \nu_{i_{m}}\in \Psi_{h_{i}}\\ i=1,2,\cdots n \end{array}}{\cup }\{\left[{\left(\frac{1}{n}\sum_{i=1}^{n}\left(\omega_{i}\nu_{i_{m}}^{qr}-\omega_{i}+1\right)\right)}^{\frac{1}{qr}}](\prod_{i=1}^{n}\frac{{\bar{p}}_{i_{m}}}{\sum_{m=1}^{\left|\Psi_{h_{i}}\right|}{\bar{p}}_{i_{m}}}\right)\}\rangle }_{q} \end{array}$$

### 4.2 q-rung orthopair probabilistic hesitant fuzzy Schweizer-Sklar geometric operator

**Definition 12.** Let $h_{i}=<\Gamma_{h_{i}},\Psi_{h_{i}}{>}_{q}(i=1,2,\cdots .n)$ be a set of q-ROPHFE, then the q-ROPHFSSG operator is defined as:

$$q-ROPHFSSG(h_{1},h_{2},\cdots ,h_{n})={\left(\overset{n}{\underset{i=1}{⊗}}h_{i}\right)}^{\frac{1}{n}}$$

**Theorem 9.** Let $h_{i}=<\Gamma_{h_{i}},\Psi_{h_{i}}{>}_{q}(i=1,2,\cdots .n)$ be a set of q-ROPHFE, then the result of using q-ROPHFSSG operator to aggregate information is still the q-ROPHFE, and satisfies:

$$\begin{array}{l} q-ROPHFSSG(h_{1},h_{2},\cdots ,h_{n})=\\ {\langle \underset{\begin{array}{c} \mu_{i_{l}}\in \Gamma_{h_{i}}\\ i=1,2,\cdots n \end{array}}{\cup }\{\left[{\left(\frac{1}{n}\sum_{i=1}^{n}\mu_{i_{l}}^{qr}\right)}^{\frac{1}{qr}}](\prod_{i=1}^{n}\frac{p_{i_{l}}}{\sum_{l=1}^{\left|\Gamma_{h_{i}}\right|}p_{i_{l}}}\right|\},\underset{\begin{array}{l} \nu_{i_{m}}\in \Psi_{h_{i}}\\ i=1,2,\cdots n \end{array}}{\cup }\{\left[{\left(1-{\left(\frac{1}{n}\sum_{i=1}^{n}{\left(1-\nu_{i_{m}}^{q}\right)}^{r}\right)}^{\frac{1}{r}}\right]}^{\frac{1}{q}})(\prod_{i=1}^{n}\frac{{\bar{p}}_{i_{m}}}{\sum_{m=1}^{\left|\Psi_{h_{i}}\right|}{\bar{p}}_{i_{m}}}\right)\}\rangle }_{q} \end{array}$$

Similar to the q-ROPHFSSA operator, the q-ROPHFSSG operator also satisfies the properties of idempotence, monotonicity, boundedness, and permutation invariance.

**Definition 13.** Let $h_{i}=<\Gamma_{h_{i}},\Psi_{h_{i}}{>}_{q}(i=1,2,\cdots .n)$ be a set of q-ROPHFE, the weight vector is ***ω*** = (*ω*_1_,*ω*_2_,⋯,*ω*_*n*_)^*T*^, and satisfies $\sum_{i=1}^{n}\omega_{i}=1$ and *ω*_*i*_∈[0,1], then the q-rung orthopair probabilistic hesitant fuzzy Schweizer-Sklar weighted geometric (q-ROPHFSSWG) operator is defined as:

$$q-ROPHFSSWG(h_{1},h_{2},\cdots ,h_{n})={\left(\overset{n}{\underset{i=1}{⊗}}h_{i}^{w_{i}}\right)}^{\frac{1}{n}}$$

**Theorem 10.** Let $h_{i}=<\Gamma_{h_{i}},\Psi_{h_{i}}{>}_{q}(i=1,2,\cdots .n)$ be a set of q-ROPHFE, the weight vector is ***ω*** = (*ω*_1_,*ω*_2_,⋯,*ω*_*n*_)^*T*^, and satisfies $\sum_{i=1}^{n}\omega_{i}=1$ and *ω*_*i*_∈[0,1], then the result of using q-ROPHFSSWG operator to aggregate information is still the q-ROPHFE, and satisfies:

$$\begin{array}{l} q-ROPHFSSWG(h_{1},h_{2},\cdots ,h_{n})=\\ {\langle \underset{\begin{array}{c} \mu_{i_{l}}\in \Gamma_{h_{i}}\\ i=1,2,\cdots n \end{array}}{\cup }\{\left[{\left(\frac{1}{n}\sum_{i=1}^{n}\left(\omega_{i}\mu_{i_{l}}^{qr}-\omega_{i}+1\right)\right)}^{\frac{1}{qr}}](\prod_{i=1}^{n}\frac{p_{i_{l}}}{\sum_{l=1}^{\left|\Gamma_{h_{i}}\right|}p_{i_{l}}}\right)\},\underset{\begin{array}{l} \nu_{i_{m}}\in \Psi_{h_{i}}\\ i=1,2,\cdots n \end{array}}{\cup }\{\left[{\left(1-{\left(\frac{1}{n}\sum_{i=1}^{n}\left(\omega_{i}{\left(1-\nu_{i_{m}}^{q}\right)}^{r}-\omega_{i}+1\right)\right)}^{\frac{1}{r}}\right)}^{\frac{1}{q}}](\prod_{i=1}^{n}\frac{{\bar{p}}_{i_{m}}}{\sum_{m=1}^{\left|\Psi_{h_{i}}\right|}{\bar{p}}_{i_{m}}}\right)\}\rangle }_{q} \end{array}$$

### 4.3 q-rung orthopair probabilistic hesitant fuzzy Schweizer-Sklar Hamy mean operator

**Definition 14.** Let *k*>0, $h_{i}=<\Gamma_{h_{i}},\Psi_{h_{i}}{>}_{q}(i=1,2,\cdots .n)$ be a set of q-ROPHFE, then the q-ROPHFSSHM operator is defined as:

$$q-ROPHFSSHM(h_{1},h_{2},\cdots ,h_{n})=\frac{\underset{\partial \in S_{n}}{⊕}{\left(\overset{k}{\underset{i=1}{⊗}}h_{\partial (i)}\right)}^{\frac{1}{k}}}{C_{n}^{k}}$$

Where ∂ = (∂(1),∂(2),⋯,∂(*n*)) represents all k-element combinations traversing (1,2,⋯,*n*), and satisfies 1≤∂(1)<⋯<∂(*n*), *S*_*n*_ is the set of all possible ∂, and $C_{n}^{k}$ represents the binomial coefficient.

**Theorem 11.** Let *k*>0, $h_{i}=<\Gamma_{h_{i}},\Psi_{h_{i}}{>}_{q}(i=1,2,\cdots .n)$ be a set of q-ROPHFE, then the result of using q-ROPHFSSHM operator to aggregate information is still the q-ROPHFE, and satisfies:

$$\begin{array}{l} q-ROPHFSSHM(h_{1},h_{2},\cdots ,h_{n})=\\ \langle \underset{\begin{array}{c} \mu_{\partial {\left(i\right)}_{l}}\in \Gamma_{h_{\partial (i)}}\\ i=1,2,\cdots k \end{array}}{\cup }\{\left[{\left(1-{\left(\frac{1}{C_{n}^{k}}\sum_{\partial \in S_{n}}{\left(1-{\left(\frac{1}{k}\sum_{i=1}^{k}\mu_{\partial {\left(i\right)}_{l}}^{qr}\right)}^{\frac{1}{r}}\right)}^{r}\right)}^{\frac{1}{r}}\right)}^{\frac{1}{q}}](\prod_{\partial \in S_{n}}\frac{\prod_{i=1}^{k}p_{\partial {\left(i\right)}_{l}}}{\prod_{i=1}^{k}\sum_{l=1}^{\left|\Gamma_{h_{\partial (i)}}\right|}p_{\partial {\left(i\right)}_{l}}}\right)\},\\ {\underset{\begin{array}{c} \nu_{\partial {\left(i\right)}_{m}}\in \Psi_{h_{\partial (i)}}\\ i=1,2,\cdots k \end{array}}{\cup }\{\left[{\left(\frac{1}{C_{n}^{k}}\sum_{\partial \in S_{n}}{\left(1-{\left(\frac{1}{k}\sum_{i=1}^{k}{\left(1-\nu_{\partial {\left(i\right)}_{m}}^{q}\right)}^{r}\right)}^{\frac{1}{r}}\right)}^{r}\right)}^{\frac{1}{qr}}](\prod_{\partial \in S_{n}}\frac{\prod_{i=1}^{k}{\bar{p}}_{\partial {\left(i\right)}_{m}}}{\prod_{i=1}^{k}\sum_{m=1}^{\left|\Psi_{h_{\partial (i)}}\right|}{\bar{p}}_{\partial {\left(i\right)}_{m}}}\right)\}\rangle }_{q} \end{array}$$

**Prove:** Firstly, use mathematical induction to prove:

$$\overset{k}{\underset{i=1}{⊗}}h_{\partial (i)}={\langle \underset{\begin{array}{c} \mu_{\partial {\left(i\right)}_{l}}\in \Gamma_{h_{\partial (i)}}\\ i=1,2,\cdots k \end{array}}{\cup }\{\left[{\left(\sum_{i=1}^{k}\mu_{\partial {\left(i\right)}_{l}}^{qr}-k+1\right)}^{\frac{1}{qr}}](\frac{\prod_{i=1}^{k}p_{\partial {\left(i\right)}_{l}}}{\prod_{i=1}^{k}\sum_{l=1}^{\left|\Gamma_{h_{\partial (i)}}\right)}p_{\partial {\left(i\right)}_{l}}}\right)\},\underset{\begin{array}{c} \nu_{\partial {\left(i\right)}_{m}}\in \Psi_{h_{\partial (i)}}\\ i=1,2,\cdots k \end{array}}{\cup }\{\left[{\left(1-{\left(\sum_{i=1}^{k}{\left(1-\nu_{\partial {\left(i\right)}_{m}}^{q}\right)}^{r}-k+1\right)}^{\frac{1}{r}}\right)}^{\frac{1}{q}}](\frac{\prod_{i=1}^{k}{\bar{p}}_{\partial {\left(i\right)}_{m}}}{\prod_{i=1}^{k}\sum_{m=1}^{\left|\Psi_{h_{\partial (i)}}\right)}{\bar{p}}_{\partial {\left(i\right)}_{m}}}\right)\}\rangle }_{q}$$

By definition 7, we can get:

$${\left(\overset{k}{\underset{i=1}{⊗}}h_{\partial (i)}\right)}^{\frac{1}{k}}={\langle \underset{\begin{array}{c} \mu_{\partial {\left(i\right)}_{l}}\in \Gamma_{h_{\partial (i)}}\\ i=1,2,\cdots k \end{array}}{\cup }\{\left[{\left(\frac{1}{k}\sum_{i=1}^{k}\mu_{\partial {\left(i\right)}_{l}}^{qr}\right)}^{\frac{1}{qr}}](\frac{\prod_{i=1}^{k}p_{\partial {\left(i\right)}_{l}}}{\prod_{i=1}^{k}\sum_{l=1}^{\left|\Gamma_{h_{\partial (i)}}\right)}p_{\partial {\left(i\right)}_{l}}}\right)\},\underset{\begin{array}{c} \nu_{\partial {\left(i\right)}_{m}}\in \Psi_{h_{\partial (i)}}\\ i=1,2,\cdots k \end{array}}{\cup }\{\left[{\left(1-{\left(\frac{1}{k}\sum_{i=1}^{k}{\left(1-\nu_{\partial {\left(i\right)}_{m}}^{q}\right)}^{r}\right)}^{\frac{1}{r}}\right)}^{\frac{1}{q}}](\frac{\prod_{i=1}^{k}{\bar{p}}_{\partial {\left(i\right)}_{m}}}{\prod_{i=1}^{k}\sum_{m=1}^{\left|\Psi_{h_{\partial (i)}}\right)}{\bar{p}}_{\partial {\left(i\right)}_{m}}}\right)\}\rangle }_{q}$$

Because of $\sum_{i=1}^{k}\frac{\prod_{i=1}^{k}p_{\partial {\left(i\right)}_{l}}}{\prod_{i=1}^{k}\sum_{l=1}^{\left|\Gamma_{h_{\partial (i)}}\right|}p_{\partial {\left(i\right)}_{l}}}=1$, $\sum_{i=1}^{k}\frac{\prod_{i=1}^{k}{\bar{p}}_{\partial {\left(i\right)}_{m}}}{\prod_{i=1}^{k}\sum_{m=1}^{\left|\Psi_{h_{\partial (i)}}\right|}{\bar{p}}_{\partial {\left(i\right)}_{m}}}=1$, it can be obtained by mathematical induction:

$$\begin{array}{l} \underset{\partial \in S_{n}}{⊕}{\left(\overset{k}{\underset{i=1}{⊗}}h_{\partial (i)}\right)}^{\frac{1}{k}}=\\ \langle \underset{\begin{array}{c} \mu_{\partial {\left(i\right)}_{l}}\in \Gamma_{h_{\partial (i)}}\\ i=1,2,\cdots k \end{array}}{\cup }\{\left[{\left(1-{\left(\sum_{\partial \in S_{n}}{\left(1-{\left(\frac{1}{k}\sum_{i=1}^{k}\mu_{\partial {\left(i\right)}_{l}}^{qr}\right)}^{\frac{1}{r}}\right)}^{r}-C_{n}^{k}+1\right)}^{\frac{1}{r}}\right)}^{\frac{1}{q}}](\prod_{\partial \in S_{n}}\frac{\prod_{i=1}^{k}p_{\partial {\left(i\right)}_{l}}}{\prod_{i=1}^{k}\sum_{l=1}^{\left|\Gamma_{h_{\partial (i)}}\right|}p_{\partial {\left(i\right)}_{l}}}\right)\},\\ {\underset{\begin{array}{c} \nu_{\partial {\left(i\right)}_{m}}\in \Psi_{h_{\partial (i)}}\\ i=1,2,\cdots k \end{array}}{\cup }\{\left[{\left(\sum_{\partial \in S_{n}}{\left(1-{\left(\frac{1}{k}\sum_{i=1}^{k}{\left(1-\nu_{\partial {\left(i\right)}_{m}}^{q}\right)}^{r}\right)}^{\frac{1}{r}}\right)}^{r}-C_{n}^{k}+1\right)}^{\frac{1}{qr}}](\prod_{\partial \in S_{n}}\frac{\prod_{i=1}^{k}{\bar{p}}_{\partial {\left(i\right)}_{m}}}{\prod_{i=1}^{k}\sum_{m=1}^{\left|\Psi_{h_{\partial (i)}}\right|}{\bar{p}}_{\partial {\left(i\right)}_{m}}}\right)\}\rangle }_{q} \end{array}$$

Continuing from definition 7, we can get:

$$\begin{array}{l} \frac{\underset{\partial \in S_{n}}{⊕}{\left(\overset{k}{\underset{i=1}{⊗}}h_{\partial (i)}\right)}^{\frac{1}{k}}}{C_{n}^{k}}=\\ \langle \underset{\begin{array}{c} \mu_{\partial {\left(i\right)}_{l}}\in \Gamma_{h_{\partial (i)}}\\ i=1,2,\cdots k \end{array}}{\cup }\{\left[{\left(1-{\left(\frac{1}{C_{n}^{k}}\sum_{\partial \in S_{n}}{\left(1-{\left(\frac{1}{k}\sum_{i=1}^{k}\mu_{\partial {\left(i\right)}_{l}}^{qr}\right)}^{\frac{1}{r}}\right)}^{r}\right)}^{\frac{1}{r}}\right)}^{\frac{1}{q}}](\prod_{\partial \in S_{n}}\frac{\prod_{i=1}^{k}p_{\partial {\left(i\right)}_{l}}}{\prod_{i=1}^{k}\sum_{l=1}^{\left|\Gamma_{h_{\partial (i)}}\right|}p_{\partial {\left(i\right)}_{l}}}\right)\},\\ {\underset{\begin{array}{c} \nu_{\partial {\left(i\right)}_{m}}\in \Psi_{h_{\partial (i)}}\\ i=1,2,\cdots k \end{array}}{\cup }\{\left[{\left(\frac{1}{C_{n}^{k}}\sum_{\partial \in S_{n}}{\left(1-{\left(\frac{1}{k}\sum_{i=1}^{k}{\left(1-\nu_{\partial {\left(i\right)}_{m}}^{q}\right)}^{r}\right)}^{\frac{1}{r}}\right)}^{r}\right)}^{\frac{1}{qr}}](\prod_{\partial \in S_{n}}\frac{\prod_{i=1}^{k}{\bar{p}}_{\partial {\left(i\right)}_{m}}}{\prod_{i=1}^{k}\sum_{m=1}^{\left|\Psi_{h_{\partial (i)}}\right|}{\bar{p}}_{\partial {\left(i\right)}_{m}}}\right)\}\rangle }_{q} \end{array}$$

That is, theorem 11 is proved.

The q-ROPHFSSHM operator has the following properties:

**Theorem 12. (Idempotence)** Let *k*>0, $h_{i}=<\Gamma_{h_{i}},\Psi_{h_{i}}{>}_{q}(i=1,2,\cdots .n)$ be a set of q-ROPHFE, if there is *h*_*i*_ = *h* for any *i*(*i* = 1,2,⋯.*n*), then:

$$q-ROPHFSSHM(h_{1},h_{2},\cdots ,h_{n})=h$$

**Theorem 13. (Monotonicity)** Let *k*>0, $h_{i}=<\Gamma_{h_{i}},\Psi_{h_{i}}{>}_{q}(i=1,2,\cdots .n)$, $h_{i}^{\prime }=<\Gamma_{h_{i}^{\prime }},\Psi_{h_{i}^{\prime }}{>}_{q}(i=1,2,\cdots .n)$ be two sets of q-ROPHFE, if there is $h_{i}<h_{i}^{\prime }$ for any *i*(*i* = 1,2,⋯.*n*), then:

$$q-ROPHFSSHM(h_{1},h_{2},\cdots ,h_{n})\leq q-ROPHFSSHM(h_{1}^{\prime },h_{2}^{\prime },\cdots ,h_{n}^{\prime })$$

**Theorem 14. (Boundedness)** Let *k*>0, $h_{i}=<\Gamma_{h_{i}},\Psi_{h_{i}}{>}_{q}(i=1,2,\cdots .n)$ be a set of q-ROPHFE, $h^{-}=<\Gamma_{h}^{-},\Psi_{h}^{+}{>}_{q}$, $h^{+}=<\Gamma_{h}^{+},\Psi_{h}^{-}{>}_{q}$, where *μ*^−^ = min(*μ*∈Γ_*i*_}, *μ*^+^ = max(*μ*∈Γ_*i*_}, *ν*^−^ = min(*ν*∈Ψ_*i*_}, *ν*^+^ = max(*ν*∈Ψ_*i*_}, then:

$$h^{-}\leq q-ROPHFSSHM(h_{1},h_{2},\cdots ,h_{n})\leq h^{+}$$

**Theorem 15. (Permutation invariance)** Let *k*>0, $h_{i}=<\Gamma_{h_{i}},\Psi_{h_{i}}{>}_{q}(i=1,2,\cdots .n)$ be a set of q-ROPHFE, For any permutation of *h*_*i*_(*i* = 1,2,⋯.*n*) and $h_{i}^{\prime }(i=1,2,\cdots .n)$, there are:

$$q-ROPHFSSHM(h_{1},h_{2},\cdots ,h_{n})=q-ROPHFSSHM(h_{1}^{\prime },h_{2}^{\prime },\cdots ,h_{n}^{\prime })$$

By assigning different parameters to *k*, the following two special cases can be obtained:

**Situation 1.** If *k* = 1, based on the definition of q-ROPHFSSHM operator, then q-ROPHFSSHM operator degenerates into the q-ROPHFSSA operator:

$$\begin{array}{l} q-ROPHFSSHM(h_{1},h_{2},\cdots ,h_{n})=\frac{1}{n}\underset{\partial \in S_{n}}{⊕}h_{\partial (i)}=\\ {\langle \underset{\begin{array}{l} \mu_{i_{l}}\in \Gamma_{h_{i}}\\ i=1,2,\cdots n \end{array}}{\cup }\{\left[{\left(1-{\left(\frac{1}{n}\sum_{i=1}^{n}{\left(1-\mu_{i_{l}}^{q}\right)}^{r}\right)}^{\frac{1}{r}}\right)}^{\frac{1}{q}}](\frac{\prod_{i=1}^{n}p_{i_{l}}}{\prod_{i=1}^{n}\sum_{l=1}^{\left|\Gamma_{h_{i}}\right|}p_{i_{l}}}\right)\},\underset{\begin{array}{l} \nu_{i_{m}}\in \Psi_{h_{i}}\\ i=1,2,\cdots n \end{array}}{\cup }\{\left[{\left(\frac{1}{n}\sum_{i=1}^{n}\nu_{i_{m}}^{qr}\right)}^{\frac{1}{qr}}](\frac{\prod_{i=1}^{n}{\bar{p}}_{i_{m}}}{\prod_{i=1}^{n}\sum_{m=1}^{\left|\Psi_{h_{i}}\right|}{\bar{p}}_{i_{m}}}\right)\}\rangle }_{q}\\ =q-ROPHFSSA(h_{1},h_{2},\cdots ,h_{n}) \end{array}$$

**Situation 2.** If *k* = *n*, based on the definition of q-ROPHFSSHM operator, then q-ROPHFSSHM operator degenerates into the q-ROPHFSSG operator:

$$\begin{array}{l} q-ROPHFSSHM(h_{1},h_{2},\cdots ,h_{n})={\left(\overset{n}{\underset{i=1}{⊗}}h_{\partial (i)}\right)}^{\frac{1}{n}}=\\ {\langle \underset{\begin{array}{l} \mu_{i_{l}}\in \Gamma_{h_{i}}\\ i=1,2,\cdots n \end{array}}{\cup }\{\left[{\left(\frac{1}{n}\sum_{i=1}^{n}\mu_{i_{l}}^{qr}\right)}^{\frac{1}{qr}}](\frac{\prod_{i=1}^{n}p_{i_{l}}}{\prod_{i=1}^{n}\sum_{l=1}^{\left|\Gamma_{h_{i}}\right|}p_{i_{l}}}\right)\},\underset{\begin{array}{l} \nu_{i_{m}}\in \Psi_{h_{i}}\\ i=1,2,\cdots n \end{array}}{\cup }\{\left[{\left(1-{\left(\frac{1}{n}\sum_{i=1}^{n}{\left(1-\nu_{i_{m}}^{q}\right)}^{r}\right)}^{\frac{1}{r}}\right)}^{\frac{1}{q}}](\frac{\prod_{i=1}^{n}{\bar{p}}_{i_{m}}}{\prod_{i=1}^{n}\sum_{m=1}^{\left|\Psi_{h_{i}}\right|}{\bar{p}}_{i_{m}}}\right)\}\rangle }_{q}\\ =q-ROPHFSSG(h_{1},h_{2},\cdots ,h_{n}) \end{array}$$

In actual problems, the degree of influence of different attributes is often different, and there are extreme values in the data. For this reason, considering the weight influence between attributes and the support relationship, a q-rung orthopair probabilistic hesitant fuzzy Schweizer-Sklar power weighted Hamy mean operator is proposed.

**Definition 15.** Let *k*>0, $h_{i}=<\Gamma_{h_{i}},\Psi_{h_{i}}{>}_{q}(i=1,2,\cdots .n)$ be a set of q-ROPHFE, the weight vector is ***ω*** = (*ω*_1_,*ω*_2_,⋯,*ω*_*n*_)^*T*^, and satisfies $\sum_{i=1}^{n}\omega_{i}=1$ and *ω*_*i*_∈[0,1], then the q-ROPHFSSPWHM operator is defined as:

$$q-ROPHFSSPWHM(h_{1},h_{2},\cdots ,h_{n})=\frac{\underset{\partial \in S_{n}}{⊕}{\left(\overset{k}{\underset{i=1}{⊗}}h_{\partial (i)}^{\varpi_{\partial (i)}}\right)}^{\frac{1}{k}}}{C_{n}^{k}}$$

Where $\varpi_{\partial (i)}=\frac{\omega_{\partial (i)}(1+T(h_{\partial (i)}))}{\sum_{j=1}^{n}\omega_{j}(1+T(h_{j}))}$, $T(h_{i})=\sum_{j=1,j\neq i}^{n}Sup(h_{i},h_{j})$, *Sup*(*h*_*i*_,*h*_*j*_) = 1−*d*(*h*_*i*_,*h*_*j*_).

**Theorem 16.** Let *k*>0, $h_{i}=<\Gamma_{h_{i}},\Psi_{h_{i}}{>}_{q}(i=1,2,\cdots .n)$ be a set of q-ROPHFE, the weight vector is ***ω*** = (*ω*_1_,*ω*_2_,⋯,*ω*_*n*_)^*T*^, and satisfies $\sum_{i=1}^{n}\omega_{i}=1$ and *ω*_*i*_∈[0,1], then the result of using q-ROPHFSSWHM operator to aggregate information is still the q-ROPHFE, and satisfies:

$$\begin{array}{l} q-ROPHFSSPWHM(h_{1},h_{2},\cdots ,h_{n})=\\ \langle \underset{\begin{array}{c} \mu_{\partial {\left(i\right)}_{l}}\in \Gamma_{h_{\partial (i)}}\\ i=1,2,\cdots k \end{array}}{\cup }\{\left[{\left(1-{\left(\frac{1}{C_{n}^{k}}\sum_{\partial \in S_{n}}{\left(1-{\left(\frac{1}{k}\sum_{i=1}^{k}\left(\omega_{\partial (i)}\mu_{\partial {\left(i\right)}_{l}}^{qr}-\omega_{\partial (i)}+1\right)\right)}^{\frac{1}{r}}\right)}^{r}\right)}^{\frac{1}{r}}\right)}^{\frac{1}{q}}](\prod_{\partial \in S_{n}}\frac{\prod_{i=1}^{k}p_{\partial {\left(i\right)}_{l}}}{\prod_{i=1}^{k}\sum_{l=1}^{\left|\Gamma_{h_{\partial (i)}}\right|}p_{\partial {\left(i\right)}_{l}}}\right)\},\\ {\underset{\begin{array}{c} \nu_{\partial {\left(i\right)}_{m}}\in \Psi_{h_{\partial (i)}}\\ i=1,2,\cdots k \end{array}}{\cup }\{\left[{\left(\frac{1}{C_{n}^{k}}\sum_{\partial \in S_{n}}{\left(1-{\left(\frac{1}{k}\sum_{i=1}^{k}\left(\omega_{\partial (i)}{\left(1-\nu_{\partial {\left(i\right)}_{m}}^{q}\right)}^{r}-\omega_{\partial (i)}+1\right)\right)}^{\frac{1}{r}}\right)}^{r}\right)}^{\frac{1}{qr}}](\prod_{\partial \in S_{n}}\frac{\prod_{i=1}^{k}{\bar{p}}_{\partial {\left(i\right)}_{m}}}{\prod_{i=1}^{k}\sum_{m=1}^{\left|\Psi_{h_{\partial (i)}}\right|}{\bar{p}}_{\partial {\left(i\right)}_{m}}}\right)\}\rangle }_{q} \end{array}$$

### 4.4 q-rung orthopair probabilistic hesitant fuzzy Schweizer-Sklar double Hamy mean operator

**Definition 16.** Let *k*>0, $h_{i}=<\Gamma_{h_{i}},\Psi_{h_{i}}{>}_{q}(i=1,2,\cdots .n)$ be a set of q-ROPHFE, then the q-ROPHFSSDHM operator is defined as:

$$q-ROPHFSSDHM(h_{1},h_{2},\cdots ,h_{n})={\left(\underset{\partial \in S_{n}}{⊗}\frac{\overset{k}{\underset{i=1}{⊕}}h_{\partial (i)}}{k}\right)}^{\frac{1}{C_{n}^{k}}}$$

Where ∂ = (∂(1),∂(2),⋯,∂(*n*)) represents all k-element combinations traversing (1,2,⋯*n*), and satisfies 1≤∂(1)<⋯<∂(*n*), *S*_*n*_ is the set of all possible ∂, and $C_{n}^{k}$ represents the binomial coefficient.

**Theorem 17.** Let *k*>0, $h_{i}=<\Gamma_{h_{i}},\Psi_{h_{i}}{>}_{q}(i=1,2,\cdots .n)$ be a set of q-ROPHFE, then the result of using q-ROPHFSSDHM operator to aggregate information is still the q-ROPHFE, and satisfies:

$$\begin{array}{l} q-ROPHFSSDHM(h_{1},h_{2},\cdots ,h_{n})=\\ \langle \underset{\begin{array}{c} \mu_{\partial {\left(i\right)}_{l}}\in \Gamma_{h_{\partial (i)}}\\ i=1,2,\cdots k \end{array}}{\cup }\{\left[{\left(1-{\left(\frac{1}{C_{n}^{k}}\sum_{\partial \in S_{n}}{\left(1-{\left(\frac{1}{k}\sum_{i=1}^{k}\mu_{\partial {\left(i\right)}_{l}}^{qr}\right)}^{\frac{1}{r}}\right)}^{r}\right)}^{\frac{1}{r}}\right)}^{\frac{1}{q}}](\prod_{\partial \in S_{n}}\frac{\prod_{i=1}^{k}p_{\partial {\left(i\right)}_{l}}}{\prod_{i=1}^{k}\sum_{l=1}^{\left|\Gamma_{h_{\partial (i)}}\right|}p_{\partial {\left(i\right)}_{l}}}\right)\},\\ {\underset{\begin{array}{c} \nu_{\partial {\left(i\right)}_{m}}\in \Psi_{h_{\partial (i)}}\\ i=1,2,\cdots k \end{array}}{\cup }\{\left[{\left(\frac{1}{C_{n}^{k}}\sum_{\partial \in S_{n}}{\left(1-{\left(\frac{1}{k}\sum_{i=1}^{k}{\left(1-\nu_{\partial {\left(i\right)}_{m}}^{q}\right)}^{r}\right)}^{\frac{1}{r}}\right)}^{r}\right)}^{\frac{1}{qr}}](\prod_{\partial \in S_{n}}\frac{\prod_{i=1}^{k}{\bar{p}}_{\partial {\left(i\right)}_{m}}}{\prod_{i=1}^{k}\sum_{m=1}^{\left|\Psi_{h_{\partial (i)}}\right|}{\bar{p}}_{\partial {\left(i\right)}_{m}}}\right)\}\rangle }_{q} \end{array}$$

Similar to the q-ROPHFSSHM operator, the q-ROPHFSSDHM operator also satisfies the properties of idempotence, monotonicity, boundedness, and permutation invariance.

By assigning different parameters to *k*, the following two special cases can be obtained:

**Situation 1.** If *k* = 1, based on the definition of q-ROPHFSSDHM operator, then q-ROPHFSSDHM operator degenerates into the q-ROPHFSSG operator:

$$\begin{array}{l} q-ROPHFSSDHM(h_{1},h_{2},\cdots ,h_{n})={\left(\overset{n}{\underset{i=1}{⊗}}h_{i}\right)}^{\frac{1}{n}}=\\ {\langle \underset{\begin{array}{l} \mu_{i_{l}}\in \Gamma_{h_{i}}\\ i=1,2,\cdots n \end{array}}{\cup }\{\left[{\left(\frac{1}{n}\sum_{i=1}^{n}\mu_{i_{l}}^{qr}\right)}^{\frac{1}{qr}}](\frac{\prod_{i=1}^{n}p_{i_{l}}}{\prod_{i=1}^{n}\sum_{l=1}^{\left|\Gamma_{h_{i}}\right|}p_{i_{l}}}\right)\},\underset{\begin{array}{l} \nu_{i_{m}}\in \Psi_{h_{i}}\\ i=1,2,\cdots n \end{array}}{\cup }\{\left[{\left(1-{\left(\frac{1}{n}\sum_{i=1}^{n}{\left(1-\nu_{i_{m}}^{q}\right)}^{r}\right)}^{\frac{1}{r}}\right)}^{\frac{1}{q}}](\frac{\prod_{i=1}^{n}{\bar{p}}_{i_{m}}}{\prod_{i=1}^{n}\sum_{m=1}^{\left|\Psi_{h_{i}}\right|}{\bar{p}}_{i_{m}}}\right)\}\rangle }_{q}\\ =q-ROPHFSSG(h_{1},h_{2},\cdots ,h_{n}) \end{array}$$

**Situation 2.** If *k* = *n*, based on the definition of q-ROPHFSSDHM operator, then q-ROPHFSSDHM operator degenerates into the q-ROPHFSSA operator:

$$\begin{array}{l} q-ROPHFSSDHM(h_{1},h_{2},\cdots ,h_{n})=\frac{1}{n}(\overset{n}{\underset{i=1}{⊕}}h_{i_{1}})=\\ {\langle \underset{\begin{array}{l} \mu_{i_{l}}\in \Gamma_{h_{i}}\\ i=1,2,\cdots n \end{array}}{\cup }\{\left[{\left(1-{\left(\frac{1}{n}\sum_{i=1}^{n}{\left(1-\mu_{i_{l}}^{q}\right)}^{r}\right)}^{\frac{1}{r}}\right)}^{\frac{1}{q}}](\frac{\prod_{i=1}^{n}p_{i_{l}}}{\prod_{i=1}^{n}\sum_{l=1}^{\left|\Gamma_{h_{i}}\right|}p_{i_{l}}}\right)\},\underset{\begin{array}{l} \nu_{i_{m}}\in \Psi_{h_{i}}\\ i=1,2,\cdots n \end{array}}{\cup }\{\left[{\left(\frac{1}{n}\sum_{i=1}^{n}\nu_{i_{m}}^{qr}\right)}^{\frac{1}{qr}}](\frac{\prod_{i=1}^{n}{\bar{p}}_{i_{m}}}{\prod_{i=1}^{n}\sum_{m=1}^{\left|\Psi_{h_{i}}\right|}{\bar{p}}_{i_{m}}}\right)\}\rangle }_{q}\\ =q-ROPHFSSA(h_{1},h_{2},\cdots ,h_{n}) \end{array}$$

**Definition 17.** Let *k*>0, $h_{i}=<\Gamma_{h_{i}},\Psi_{h_{i}}{>}_{q}(i=1,2,\cdots .n)$ be a set of q-ROPHFE, the weight vector is ***ω*** = (*ω*_1_,*ω*_2_,⋯,*ω*_*n*_)^*T*^, and satisfies $\sum_{i=1}^{n}\omega_{i}=1$ and *ω*_*i*_∈[0,1], then the q-ROPHFSSPWDHM operator is defined as:

$$q-ROPHFSSPWDHM(h_{1},h_{2},\cdots ,h_{n})={\left(\underset{\partial \in S_{n}}{⊗}\frac{\overset{k}{\underset{i=1}{⊕}}\varpi_{\partial (i)}h_{\partial (i)}}{k}\right)}^{\frac{1}{C_{n}^{k}}}$$

Where $\varpi_{\partial (i)}=\frac{\omega_{\partial (i)}(1+T(h_{\partial (i)}))}{\sum_{j=1}^{n}\omega_{j}(1+T(h_{j}))}$, $T(h_{i})=\sum_{j=1,j\neq i}^{n}Sup(h_{i},h_{j})$, *Sup*(*h*_*i*_,*h*_*j*_) = 1−*d*(*h*_*i*_,*h*_*j*_).

**Theorem 18** Let *k*>0, $h_{i}=<\Gamma_{h_{i}},\Psi_{h_{i}}{>}_{q}(i=1,2,\cdots .n)$ be a set of q-ROPHFE, the weight vector is ***ω*** = (*ω*_1_,*ω*_2_,⋯,*ω*_*n*_)^*T*^, and satisfies $\sum_{i=1}^{n}\omega_{i}=1$ and *ω*_*i*_∈[0,1], then the result of using q-ROPHFSSPWDHM operator to aggregate information is still the q-ROPHFE, and satisfies:

$$\begin{array}{l} q-ROPHFSSPWDHM(h_{1},h_{2},\cdots ,h_{n})=\\ \langle \underset{\begin{array}{c} \mu_{\partial {\left(i\right)}_{l}}\in \Gamma_{h_{\partial (i)}}\\ i=1,2,\cdots k \end{array}}{\cup }\{\left[{\left(\frac{1}{C_{n}^{k}}\sum_{\partial \in S_{n}}{\left(1-{\left(\frac{1}{k}\sum_{i=1}^{k}\left(\omega_{\partial (i)}{\left(1-\mu_{\partial {\left(i\right)}_{m}}^{q}\right)}^{r}-\omega_{\partial (i)}+1\right)\right)}^{\frac{1}{r}}\right)}^{r}\right)}^{\frac{1}{qr}}](\prod_{\partial \in S_{n}}\frac{\prod_{i=1}^{k}p_{\partial {\left(i\right)}_{l}}}{\prod_{i=1}^{k}\sum_{l=1}^{\left|\Gamma_{h_{\partial (i)}}\right|}p_{\partial {\left(i\right)}_{l}}}\right)\},)\\ {\underset{\begin{array}{c} \nu_{\partial {\left(i\right)}_{m}}\in \Psi_{h_{\partial (i)}}\\ i=1,2,\cdots k \end{array}}{\cup }\{\left[{\left(1-{\left(\frac{1}{C_{n}^{k}}\sum_{\partial \in S_{n}}{\left(1-{\left(\frac{1}{k}\sum_{i=1}^{k}\left(\omega_{\partial (i)}\nu_{\partial {\left(i\right)}_{l}}^{qr}-\omega_{\partial (i)}+1\right)\right)}^{\frac{1}{r}}\right)}^{r}\right)}^{\frac{1}{r}}\right)}^{\frac{1}{q}}](\prod_{\partial \in S_{n}}\frac{\prod_{i=1}^{k}{\bar{p}}_{\partial {\left(i\right)}_{m}}}{\prod_{i=1}^{k}\sum_{m=1}^{\left|\Psi_{h_{\partial (i)}}\right|}{\bar{p}}_{\partial {\left(i\right)}_{m}}}\right)\}\rangle }_{q} \end{array}$$

## 5 Multi-attribute decision-making model based on q-rung orthopair probabilistic hesitant fuzzy Schweizer-Sklar Hamy mean operator

### 5.1 Multi-attribute decision-making model

Let the set *A* = {*A*_1_,*A*_2_,⋯*A*_*m*_} composed of *m* candidate schemes of the multi-attribute decision-making model be the scheme set. The set *M* = {*M*_1_,*M*_2_,⋯,*M*_*n*_} of *n* attributes used to evaluate the scheme is the attribute set. The set ***ω*** = (*ω*_1_,*ω*_2_,⋯,*ω*_*n*_)^*T*^ is the attribute weight vector related to the attribute, and satisfies $\sum_{i=1}^{n}\omega_{i}=1$ and *ω*_*i*_∈[0,1]. The evaluation value of the scheme *A*_*i*_(*i* = 1,2,⋯,*m*) given by the decision-making expert under the attribute *M*_*j*_(*j* = 1,2,⋯*n*) can be expressed by q-ROPHFE *h*_*ij*_. After all the decision-making expert evaluate the scheme, the q-rung orthopair probabilistic hesitant fuzzy decision-making matrix ***H*** can be obtained, denoted as ***H*** = (*h*_*ij*_)_*m*×*n*_. Then the multi-attribute decision-making steps of q-rung orthopair probabilistic hesitant fuzzy Schweizer-Sklar Hamy mean operator are as follows:

Step 1: Standardize the data to obtain a standardized q-rung orthopair probabilistic hesitant fuzzy decision-making matrix ***H***. The specific processing method is as follows:

$$H^{N}={\left(h_{ij}^{N}\right)}_{m\times n}=\{\begin{array}{l} h_{ij},\;M_{ij}\;\mathrm{is}\;\mathrm{the}\;\mathrm{benefit}\;\mathrm{index}\\ {\left(h_{ij}\right)}^{c},\;M_{ij}\;\mathrm{is}\;\mathrm{the}\;\mathrm{cost}\;\mathrm{index} \end{array}$$

Step 2: To calculate the support *Sup*(*h*_*ij*_,*h*_*ik*_) between *h*_*ij*_ and *h*_*ik*_, the formula is as follows:

$$Sup(h_{ij},h_{ik})=1-d(h_{ij},h_{ik})$$

Where *i* = 1,2,⋯*m*, *j*,*k* = 1,2,⋯*n* and *k*≠*l*.

Step 3: To calculate the overall support degree *T*(*h*_*ij*_), the formula is as follows:

$$T(h_{ij})=\sum_{k=1,k\neq j}^{n}Sup(h_{ij},h_{ik})$$

Step 4: To calculate the power weight *ϖ*_*ij*_ of *h*_*ij*_, the formula is as follows:

$$\varpi_{ij}=\frac{\omega_{j}(1+T(h_{ij}))}{\sum_{j=1}^{n}\left(1+T(h_{ij})\right)}$$

Step5: Use q-rung orthopair probabilistic hesitant fuzzy Schweizer-Sklar Hamy mean operator to aggregate the evaluation data values of each scheme under different attributes, and then obtain the q-rung orthopair probabilistic hesitant fuzzy comprehensive evaluation value *h*_*i*_ corresponding to each scheme.

Step 6: Calculate the score function *S*(*h*_*i*_) and deviation *D*(*h*_*i*_) corresponding to *h*_*i*_ of each scheme, and rank them.

Step 7: Make decisions based on the ranking results.

### 5.2 Example calculation

In order to reasonably evaluate the busyness of 5 air traffic control sectors in an airport approach control area, and to provide suggestions for the development and improvement of subsequent air traffic control work. Let the set *A* = {*A*_1_,*A*_2_,*A*_3_,*A*_4_,*A*_5_} composed of 5 air traffic control control sectors of the airport is the scheme set. Let the set *M* = {*M*_1_,*M*_2_,⋯,*M*_*n*_} consisting of 4 indicators, including capacity factor (*M*_1_), airspace factor (*M*_2_), air traffic control factor (*M*_3_), and interval factor (*M*_4_) that affect the busyness of the sector, is the attribute set. Due to the different importance of different evaluation indicators, combined with expert experience, the weight vector of the above four types of indicators is given here as *ω*= (0.3,0.2,0.35,0.15)^*T*^. Collect relevant information and process it to obtain a standardized q-rung orthopair probabilistic hesitant fuzzy decision-making matrix ***H***. In order to facilitate the modeling and analysis, set the q-ROPHFE to satisfy *q* = 3, set *r* = −1 in the Schweizer-Sklar T-norm. Suppose this example uses q-ROPHFSSPWHM to aggregate information, and its parameters satisfy *k* = 2. The converted data is shown in Table 1.

**Table 1:** q-rung orthopair probability hesitant fuzzy evaluation matrix.

Scheme set	Attribute set			
	*M*_1_	*M*_2_	*M*_3_	*M*_4_
*A*_1_	〈{0.5|0.4,0.7|0.5},{0.6|0.6,0.4|0.3}〉	〈{0.4|0.3,0.7|0.6},{0.7|0.4,0.5|0.3}〉	〈{0.5|0.5,0.8|0.5},{0.4|0.6,0.3|0.3}〉	〈{0.6|0.3,0.7|0.7},{0.6|0.5,0.4|0.5}〉
*A*_2_	〈{0.5|0.8,0.6|0.1},{0.7|0.4,0.5|0.4}〉	〈{0.2|0.5,0.4|0.4},{0.7|0.4,0.6|0.5}〉	〈{0.5|0.4,0.7|0.6},{0.6|0.3,0.5|0.5}〉	〈{0.3|0.4,0.8|0.5},{0.9|0.1,0.4|0.8}〉
*A*_3_	〈{0.5|0.6,0.7|0.4},{0.6|0.7,0.5|0.3}〉	〈{0.6|0.7,0.7|0.3},{0.5|0.5,0.4|0.5}〉	〈{0.5|0.6,0.7|0.3},{0.7|0.4,0.6|0.3}〉	〈{0.6|0.4,0.8|0.3},{0.5|0.6,0.4|0.2}〉
*A*_4_	〈{0.5|0.4,0.7|0.4},{0.7|0.2,0.6|0.8}〉	〈{0.2|0.7,0.4|0.3},{0.7|0.4,0.6|0.5}〉	〈{0.6|0.5,0.7|0.4},{0.7|0.2,0.5|0.8}〉	〈{0.3|0.4,0.4|0.6},{0.8|0.3,0.7|0.5}〉
*A*_5_	〈{0.6|0.5,0.7|0.5},{0.4|0.4,0.3|0.5}〉	〈{0.5|0.7,0.9|0.1},{0.7|0.3,0.2|0.7}〉	〈{0.6|0.6,0.7|0.3},{0.6|0.4,0.5|0.6}〉	〈{0.5|0.7,0.8|0.3},{0.6|0.9,0.3|0.1}〉

The above data is normalized, and the support *Sup*(*h*_*ij*_,*h*_*ik*_) between the q-ROPHFE *h*_*ij*_ and *h*_*ik*_ is calculated. For convenience, $S_{j}^{k}=(Sup(h_{1j},h_{1k}),Sup(h_{2j},h_{2k}),\cdots Sup(h_{ij},h_{ik}))$. Where *i* = 1,2,3,4,5, *k*,*l* = 1,2,3,4 and *k*≠*l*.


$$S_{1}^{2}=S_{2}^{1}=(0.9263,0.9171,0.9233,0.8413,0.8595)$$

$$S_{1}^{3}=S_{3}^{1}=(0.9041,0.9041,0.9799,0.9574,0.9135)$$

$$S_{1}^{4}=S_{4}^{1}=(0.9487,0.8003,0.8996,0.7887,0.8522)$$

$$S_{2}^{3}=S_{3}^{2}=(0.8523,0.8212,0.9347,0.8153,0.8622)$$

$$S_{2}^{4}=S_{4}^{2}=(0.9176,0.7733,0.9639,0.9086,0.8643)$$

$$S_{3}^{4}=S_{4}^{3}=(0.8727,0.8593,0.9084,0.7786,0.9102)$$

Next, calculate the overall support *T*(*h*_*ij*_), the overall support matrix *T* = (*T*(*h*_*ij*_))_5×4_ can be obtained as follows:

$$T=[\begin{array}{cccc} 2.7791 & 2.6962 & 2.6291 & 2.7390\\ 2.6215 & 2.5116 & 2.5846 & 2.4329\\ 2.8028 & 2.8219 & 2.8230 & 2.7719\\ 2.5874 & 2.5652 & 2.5513 & 2.4759\\ 2.6252 & 2.5857 & 2.6859 & 2.6264 \end{array}]$$

Calculate the power weight *ϖ*_*ij*_ of *h*_*ij*_ and get the matrix *ϖ* = (*ϖ*_*ij*_)_5×4_:

$$\varpi =[\begin{array}{cccc} 0.3061 & 0.1996 & 0.3429 & 0.1514\\ 0.3053 & 0.1974 & 0.3526 & 0.1447\\ 0.2995 & 0.2007 & 0.3513 & 0.1485\\ 0.3029 & 0.2007 & 0.3498 & 0.1467\\ 0.2989 & 0.1971 & 0.3545 & 0.1495 \end{array}]$$

Combining the above data, using q-ROPHFSSPWHM to aggregate the data in Table 1, can get the comprehensive evaluation value of each scheme as shown in Table 2.

**Table 2:** Comprehensive evaluation value of each scheme.

Evaluation	q-ROPHFE
*h*_1_	$\begin{array}{l} \langle \{0.3338|0.0222,0.3646|0.0519,0.4584|0.0222,0.4737|0.0519,0.3866|0.0444,0.4093|0.1037,0.4856|0.0444,0.4987|0.1037,\\ 0.3976|0.0278,0.4190|0.0648,0.4198|0.0278,0.5045|0.0648,0.4351|0.0556,0.4525|0.1296,0.5146|0.0556,0.5258|0.1296\},\\ \{0.7230|0.1270,0.6789|0.1270,0.6172|0.0635,0.5927|0.0635,0.7012|0.0952,0.6618|0.0952,0.6054|0.0476,0.5826|0.0476,\\ 0.6605|0.0635,0.6289|0.0635,0.5818|0.0317,0.5622|0.0317,0.6451|0.0476,0.6162|0.0476,0.5724|0.0238,0.5540|0.0238\}\rangle \end{array}$
*h*_2_	$\begin{array}{l} \langle \{0.2831|0.0878,0.4105|0.1097,0.3662|0.1317,0.4533|0.1646,0.2946|0.0702,0.4156|0.0878,0.3729|0.1053,0.4573|0.1317,\\ 0.3178|0.0110,0.5258|0.1296,0.3872|0.0165,0.4661|0.0206,0.3269|0.0088,0.4313|0.0110,0.3930|0.0132,0.4698|0.0165\},\\ \{0.8678|0.0093,0.7639|0.0741,0.8247|0.0154,0.7372|0.1235,0.8522|0.0116,0.7545|0.0926,0.8119|0.0193,0.7290|0.1543,\\ 0.8058|0.0093,0.7250|0.0741,0.7731|0.0154,0.7031|0.1235,0.7941|0.0116,0.7173|0.0926,0.7631|0.0193,0.6963|0.1543\}\rangle \end{array}$
*h*_3_	$\begin{array}{l} \langle \{0.3601|0.1600,0.4320|0.1200,0.4157|0.0800,0.4703|0.0600,0.3866|0.0686,0.4496|0.0514,0.4351|0.0343,0.4846|0.0257,\\ 0.4157|0.1067,0.4703|0.0800,0.4575|0.0533,0.5017|0.0400,0.4351|0.0457,0.4846|0.0343,0.4728|0.0229,0.5138|0.0171\},\\ \{0.7789|0.1500,0.7371|0.0500,0.7638|0.1125,0.7249|0.0375,0.7371|0.1500,0.7030|0.0500,0.7249|0.1125,0.6928|0.0375,\\ 0.7502|0.0643,0.7138|0.0214,0.7371|0.0482,0.7030|0.0161,0.7138|0.0643,0.6835|0.0214,0.7030|0.0482,0.6744|0.0161\}\rangle \end{array}$
*h*_4_	$\begin{array}{l} \langle \{0.3178|0.0778,0.3240|0.1167,0.3662|0.0622,0.3707|0.0933,0.3269|0.0333,0.3326|0.0500,0.3729|0.0267,0.3772|0.0400,\\ 0.3872|0.0778,0.3911|0.1167,0.4201|0.0622,0.4234|0.0933,0.3930|0.0333,0.3968|0.0500,0.4249|0.0267,0.4281|0.0400\},\\ \{0.8875|0.0067,0.8777|0.0111,0.8202|0.0267,0.8131|0.0444,0.8704|0.0083,0.8614|0.0139,0.8077|0.0333,0.8010|0.0556,\\ 0.8624|0.0267,0.8537|0.0444,0.8017|0.1067,0.7952|0.1778,0.8471|0.0333,0.8390|0.0556,0.7902|0.1333,0.7841|0.2222\}\rangle \end{array}$
*h*_5_	$\begin{array}{l} \langle \{0.3749|0.2042,0.4509|0.0875,0.4101|0.1021,0.4743|0.0438,0.5368|0.0292,0.5707|0.0125,0.5514|0.0146,0.5828|0.0063,\\ 0.4101|0.2042,0.4743|0.0875,0.4392|0.1021,0.4949|0.0438,0.5514|0.0292,0.5828|0.0125,0.5649|0.0146,0.5941|0.0063\},\\ \{0.7230|0.0480,0.6166|0.0053,0.7013|0.0720,0.6048|0.0080,0.4853|0.1120,0.4586|0.0124,0.4807|0.1680,0.4549|0.0187,\\ 0.6172|0.0600,0.5543|0.0067,0.6055|0.0900,0.5465|0.0100,0.4588|0.1400,0.4371|0.0156,0.4551|0.2100,0.4340|0.0233\}\rangle \end{array}$

According to the data in Table 2, calculate the score of each scheme, can get:

$$S(h_{1})=0.4179,\;S(h_{2})=0.3259,\;S(h_{3})=0.3332,\;S(h_{4})=0.2727,\;S(h_{5})=0.4677.$$

Rank the schemes according to the scores, and get *A*_5_≻*A*_1_≻*A*_3_≻*A*_2_≻*A*_4_, that is, the busiest air traffic control sector is *A*_5_.

#### 5.2.1 Parameter analysis

Use the q-ROPHFSSPWHM operator to aggregate information, control the parameters *q* = 3, *r* = −1, and select the ranking results when different parameters *k* are shown in Table 3.

**Table 3:** Rank results under different parameters *k*.

Parameter k	*S*(*h*_*i*_)(*i* = 1,2,3,4,5)	Rank result
*k* = 1	*S*(*h*_1_) = 0.3377, *S*(*h*_2_) = 0.2610, *S*(*h*_3_) = 0.2485, *S*(*h*_4_) = 0.2100, *S*(*h*_5_) = 0.3875	*A*_5_≻*A*_1_≻*A*_2_≻*A*_3_
*k* = 2	*S*(*h*_1_) = 0.4179, *S*(*h*_2_) = 0.3259, *S*(*h*_3_) = 0.3332, *S*(*h*_4_) = 0.2727, *S*(*h*_5_) = 0.4677	*A*_5_≻*A*_1_≻*A*_3_≻*A*_2_
*k* = 3	*S*(*h*_1_) = 0.5219, *S*(*h*_2_) = 0.4965, *S*(*h*_3_) = 0.5201, *S*(*h*_4_) = 0.4957, *S*(*h*_5_) = 0.5288	*A*_5_≻*A*_1_≻*A*_3_≻*A*_2_
*k* = 4	*S*(*h*_1_) = 0.7340, *S*(*h*_2_) = 0.5662, *S*(*h*_3_) = 0.7248, *S*(*h*_4_) = 0.5421, *S*(*h*_5_) = 0.7345	*A*_5_≻*A*_1_≻*A*_3_≻*A*_2_

From the ranking results in Table 3, the following conclusions can be drawn:

1. The evaluation results obtained under different parameter *k* values are all the busiest in air traffic control sector *A*_5_, indicating that the use of q-ROPHFSSPWHM operator for multi-attribute decision-making is reliable.
2. When *k* = 1, the ranking result is *A*_5_≻*A*_1_≻*A*_2_≻*A*_3_≻*A*_4_, which is different from the ranking results when other parameters *k* = 2, *k* = 3, and *k* = 4. This is because q-ROPHFSSPWHM operator degenerates to q-ROPHFSSA operator. The correlation between attributes cannot be well described, resulting in loss of information.
3. Use the q-ROPHFSSPWHM operator to aggregate information, control the parameters *q* = 3, *k* = 2, and select the ranking results when different parameters *r* are shown in Table 4.

**Table 4:** Rank results under different parameters *r*.

Parameter r	*S*(*h*_*i*_)(*i* = 1,2,3,4,5)	Rank result
*r* = −1	*S*(*h*_1_) = 0.4179, *S*(*h*_2_) = 0.3259, *S*(*h*_3_) = 0.3332, *S*(*h*_4_) = 0.2727, *S*(*h*_5_) = 0.4677	*A*_5_≻*A*_1_≻*A*_3_≻*A*_2_
*r* = −3	*S*(*h*_1_) = 0.5202, *S*(*h*_2_) = 0.4547, *S*(*h*_3_) = 0.4571, *S*(*h*_4_) = 0.3906, *S*(*h*_5_) = 0.5455	*A*_5_≻*A*_1_≻*A*_3_≻*A*_2_
*r* = −5	*S*(*h*_1_) = 0.5569, *S*(*h*_2_) = 0.5023, *S*(*h*_3_) = 0.5008, *S*(*h*_4_) = 0.4343, *S*(*h*_5_) = 0.5756	*A*_5_≻*A*_1_≻*A*_2_≻*A*_3_
*r* = −7	*S*(*h*_1_) = 0.5825, *S*(*h*_2_) = 0.5354, *S*(*h*_3_) = 0.5299, *S*(*h*_4_) = 0.4598, *S*(*h*_5_) = 0.5972	*A*_5_≻*A*_1_≻*A*_2_≻*A*_3_
*r* = −9	*S*(*h*_1_) = 0.6012, *S*(*h*_2_) = 0.5598, *S*(*h*_3_) = 0.5526, *S*(*h*_4_) = 0.4786, *S*(*h*_5_) = 0.6134	*A*_5_≻*A*_1_≻*A*_2_≻*A*_3_

From the ranking results in Table 4, the following conclusions can be drawn:

1. The evaluation results obtained under different parameter *r* values are all the busiest in air traffic control sector *A*_5_, which once again shows that the use of q-ROPHFSSPWHM operator for multi-attribute decision-making is reliable.
2. It can be seen from Table 4 that as the value of the parameter *r* continues to increase, the scores of each scheme are also increasing, indicating that the decision-making results are becoming more and more optimistic. In practical applications, the value of the parameter can be determined according to the style of the decision-making expert.

Use the q-ROPHFSSPWHM operator to aggregate information, control the parameters *k* = 2, *r* = −1, and select the ranking results when different parameters *q* are shown in Table 5.

**Table 5:** Rank results under different parameters *q*.

Parameter q	*S*(*h*_*i*_)(*i* = 1,2,3,4,5)	Rank result
*q* = 3	*S*(*h*_1_) = 0.4179, *S*(*h*_2_) = 0.3259, *S*(*h*_3_) = 0.3332, *S*(*h*_4_) = 0.2727, *S*(*h*_5_) = 0.4677	*A*_5_≻*A*_1_≻*A*_3_≻*A*_2_
*q* = 5	*S*(*h*_1_) = 0.4947, *S*(*h*_2_) = 0.4463, *S*(*h*_3_) = 0.4476, *S*(*h*_4_) = 0.3953, *S*(*h*_5_) = 0.5081	*A*_5_≻*A*_1_≻*A*_3_≻*A*_2_
*q* = 7	*S*(*h*_1_) = 0.5060, *S*(*h*_2_) = 0.4893, *S*(*h*_3_) = 0.4891, *S*(*h*_4_) = 0.4611, *S*(*h*_5_) = 0.5073	*A*_5_≻*A*_1_≻*A*_2_≻*A*_3_
*q* = 9	*S*(*h*_1_) = 0.5048, *S*(*h*_2_) = 0.4991, *S*(*h*_3_) = 0.4989, *S*(*h*_4_) = 0.4866, *S*(*h*_5_) = 0.5065	*A*_5_≻*A*_1_≻*A*_2_≻*A*_3_
*q* = 11	*S*(*h*_1_) = 0.5030, *S*(*h*_2_) = 0.5007, *S*(*h*_3_) = 0.5006, *S*(*h*_4_) = 0.4955, *S*(*h*_5_) = 0.5047	*A*_5_≻*A*_1_≻*A*_2_≻*A*_3_
*q* = 13	*S*(*h*_1_) = 0.5019, *S*(*h*_2_) = 0.5006, *S*(*h*_3_) = 0.5005, *S*(*h*_4_) = 0.4985, *S*(*h*_5_) = 0.5028	*A*_5_≻*A*_1_≻*A*_2_≻*A*_3_
*q* = 15	*S*(*h*_1_) = 0.5010, *S*(*h*_2_) = 0.5004, *S*(*h*_3_) = 0.5003, *S*(*h*_4_) = 0.4995, *S*(*h*_5_) = 0.5012	*A*_5_≻*A*_1_≻*A*_2_≻*A*_3_
*q* = 17	*S*(*h*_1_) = 0.5006, *S*(*h*_2_) = 0.5003, *S*(*h*_3_) = 0.5002, *S*(*h*_4_) = 0.4998, *S*(*h*_5_) = 0.5008	*A*_5_≻*A*_1_≻*A*_2_≻*A*_3_
*q* = 19	*S*(*h*_1_) = 0.5004, *S*(*h*_2_) = 0.5002, *S*(*h*_3_) = 0.5001, *S*(*h*_4_) = 0.4999, *S*(*h*_5_) = 0.5006	*A*_5_≻*A*_1_≻*A*_2_≻*A*_3_

From the ranking results in Table 5, the following conclusions can be drawn:

1. The evaluation results obtained under different parameter *q* values are all the busiest in air traffic control sector *A*_5_, it further shows that the use of q-ROPHFSSPWHM operator for multi-attribute decision-making has better robustness.
2. As the value of the parameter *q* increases, the score function of each scheme first increases and then decreases, and finally tends to 0.5. This is because as the value of the parameter increases, the degree of support between the hesitant fuzzy elements tends to 1, which makes the q-ROPHFSSPWHM operator useless. Therefore, in practical applications, the value of q should be selected reasonably to avoid the adverse effects caused by the above situations.

#### 5.2.2 Comparative analysis

The following compares the q-ROPHFSSPWHM operator proposed in this paper with the operators proposed in documents [19,31,35–37] and calculates the ranking results, as shown in Table 6.

**Table 6:** Rank results of different operators.

operator	*S*(*h*_*i*_)(*i* = 1,2,3,4,5)	Rank result
IVPHFFA [31]	*S*(*h*_1_) = 0.3707, *S*(*h*_2_) = 0.3201, *S*(*h*_3_) = 0.3564, *S*(*h*_4_) = 0.3082, *S*(*h*_5_) = 0.3872	*A*_5_≻*A*_1_≻*A*_2_≻*A*_3_
PyPHFHWA [35]	*S*(*h*_1_) = 0.3405, *S*(*h*_2_) = 0.2715, *S*(*h*_3_) = 0.2838, *S*(*h*_4_) = 0.2564, *S*(*h*_5_) = 0.3621	*A*_5_≻*A*_1_≻*A*_2_≻*A*_3_
q-ROPHFPWMM [19]	*S*(*h*_1_) = 0.3894, *S*(*h*_2_) = 0.3114, *S*(*h*_3_) = 0.3361, *S*(*h*_4_) = 0.2476, *S*(*h*_5_) = 0.4223	*A*_5_≻*A*_1_≻*A*_3_≻*A*_2_
q-ROHFPGHM [36]	*S*(*h*_1_) = 0.4861, *S*(*h*_2_) = 0.4554, *S*(*h*_3_) = 0.3876, *S*(*h*_4_) = 0.3861, *S*(*h*_5_) = 0.4223	*A*_1_≻*A*_2_≻*A*_5_≻*A*_3_
q-RPDHFPWMM [37]	*S*(*h*_1_) = 0.5000, *S*(*h*_2_) = 0.5000, *S*(*h*_3_) = 0.5000, *S*(*h*_4_) = 0.0500, *S*(*h*_5_) = 0.5000	Indistinction
q-ROPHFSSPWHM	*S*(*h*_1_) = 0.4179, *S*(*h*_2_) = 0.3259, *S*(*h*_3_) = 0.3332, *S*(*h*_4_) = 0.2727, *S*(*h*_5_) = 0.4677	*A*_5_≻*A*_1_≻*A*_3_≻*A*_2_

From the ranking results in Table 6, the following conclusions can be drawn.

It can be seen from the sorting results that the IVPHFFA operator, PyPHFHWA operator, and q-ROPHFPWMM operator are in the same order, but IVPHFFA operator and PyPHFHWA operator do not meet the data constraints, and the scope of application is limited. The q-ROPHFPWMM operator operator is not flexible enough to make decisions according to the multiple preferences and habits of decision makers. The sorting of the q-RPDHFPWMM operator is inconsistent with the sorting of other operators, mainly because the probability information of the data is not considered, and there is information loss. The q-ROPHFSSPWHM operator, although the probability information is considered, is not normalized in the information integration, resulting in a close score function, and it is impossible to distinguish the pros and cons of the scheme. In addition, compared with the PLqROFWGDBM operator [38] with linguistic variables, the operator proposed in this paper is more convenient in data processing. For the PHFWA operator [39], it considers the non-membership information and more comprehensively describes the evaluation information of the decision makers.

## 6 Conclusions

In order to further improve the computing power of the information integration operator in the q-rung orthopair probabilistic hesitant fuzzy environment, this paper proposes a multi-attribute decision-making method based on the q-ROPHFSSDHM operator. The reliability and validity of the research content in this paper are verified through decision-making examples and comparative analysis. The main contributions are as follows:

1. Based on Schweizer-Sklar T-norm, the operation rules of q-rung orthopair probabilistic hesitant fuzzy set are defined, which can provide reference for related research.
2. A new q-rung orthopair probabilistic hesitant fuzzy distance measure is proposed. This distance measure introduces the degree of hesitation, which more reasonably reflects the different opinions of decision-making expert, and helps to measure the distance of the q-ROPHFE more reasonably.
3. The q-ROPHFSSHM operator, the q-ROPHFSSDHM operator and their power weighted form are proposed. The above operators fully consider the correlation between attributes and the influence of their importance to make the decision result more reasonable.

Through the work and research of this paper, it is found that there are still some deficiencies and limitations in the research of this paper. The probability information in this paper is discrete, and the continuous probability information may be more in line with the actual situation. Therefore, in the follow-up work, further research on probability Cinformation is needed. In order to further characterize the correlation between attributes, the next step will be to study the Muirhead mean aggregation operator [40] and the similarity-distance measures [41].
